# Unmet supportive care needs of head and neck cancer survivors: A scoping review

**DOI:** 10.1371/journal.pone.0347295

**Published:** 2026-04-15

**Authors:** Ya Huang, Lan Chen, Mi Zeng, Jingjing Wu, Yan Lu, Huiru Zhang, Xiaoshan Li, Huijun Quan, Guqing Zeng

**Affiliations:** 1 School of Nursing, The First Affiliated Hospital, Hengyang Medical School, University of South China, Hengyang, Hunan, China; 2 Wuxi 9th People’s Hospital Affiliated to Suzhou University, Wuxi, Jiangsu, China; Ankara University, TÜRKIYE

## Abstract

**Aim:**

To investigate the unmet supportive care needs, the existing tools to screen these unmet needs, and the factors associated with them among head and neck cancer survivors.

**Methods:**

A systematic search was conducted across eleven databases, including Web of Science and PubMed. The search covered the period from the inception of each database up to August 20, 2025. Identified records were screened for relevance, followed by data extraction and analysis.

**Results:**

A total of 4074 articles were identified, of which 12 were included. 7 assessment tools were identified. Among these, 3 offered a relatively comprehensive scope, 2 focused specifically on disease-functional needs, and 1 incorporated the disease -lifestyle needs. Head and neck cancer survivors reported unmet supportive care needs across health system/information, psychological, patient care/support, physical/daily living, sexuality, disease-specific functioning and disease-specific life. Health system/information, patient care/support, psychological needs are the top three unmet needs. Some demographic, clinical, and psychological factors are associated with these unmet needs.

**Conclusion:**

Head and neck cancer survivors experience considerable unmet supportive care needs in health system/information, psychological and patient care/support. Existing assessment tools lack comprehensiveness, failing to integrate both universal and disease-specific needs. Future development of a multidimensional, integrated tool is essential. Such instruments will enable multidisciplinary teams to deliver personalized support care informed by assessment results.

## 1. Introduction

Head and neck cancer (HNC) includes cancers of the sinus, nasopharynx, oro and hypopharynx, larynx, oral cavity and salivary glands. It ranks as the world’s sixth most common cancer category, accounting for approximately 5% of all reported cancer cases [1]. Global incidence rate has risen in recent decades [2],and is projected to increase by 30% by 2030 [3,4]. This trend is evident across both developed and developing countries [5]. With the development and introduction of effective treatment options for HNC [6,7], considerable improvements in HNC survival rates have been observed in recent years [8]. As growing numbers of HNC patients transition into survivorship, their persistent health challenges and quality-of-life concerns demand increased clinical attention.

The primary treatment for HNC involves surgery and radiotherapy, often combined with chemotherapy. Although essential for survival, the treatment for HNC often result in severe and long-term morbidity. Surgical resection can cause significant alterations in appearance and impairments in core functions such as swallowing, speech, and breathing. Radiotherapy frequently leads to permanent tissue damage, resulting in debilitating sequelae including dysphagia, tissue fibrosis, and pain [9]. When used as a radiosensitizer, chemotherapy introduces systemic toxicities such as ototoxicity, nephrotoxicity, and significant gastrointestinal events [10]. These adverse effects impair the quality of life of HNC survivors during post-treatment survivorship [11]. Furthermore, HNC survivors have an increased risk of developing secondary primary cancers (SPCs), particularly in the head and neck region and lungs [12]. Consequently, HNC survivors often experience complex supportive care needs related to treatment sequelae, SPC risks, and significant physical and psychosocial rehabilitation [12–15].

Although the multifaceted supportive care needs of HNC survivors are well-established, unmet supportive care needs persistently afflict HNC survivors as documented in prior research [13,14,16]. Unmet supportive care needs persistently impact HNC survivors, adversely affecting functional recovery and long-term well-being. Nevertheless, significant knowledge gaps remain regarding the comprehensive profile of these unmet needs and their determinants. To address this deficit and establish targeted priorities for clinical interventions and future research, this study will conduct a scoping review. The primary objectives of this study are: (1) to systematically identify and categorize core unmet supportive care needs in post-treatment HNC survivors; (2) to determine key factors associated with these unmet needs.

## 2. Methods

### 2.1. Review framework

This scoping review was conducted using the methodological framework by Arksey and O’Malley [17,18] and reported following the PRISMA-ScR guidelines (S1 File). The study was prospectively registered on the Open Science Framework (https://doi.org/10.17605/OSF.IO/YVA2W).

### 2.2. Review questions

(1) What are the unmet supportive care needs in HNC survivors? (2) What assessment tools are available to evaluate unmet supportive care needs in HNC survivors? (3) What factors associated with unmet supportive care needs in HNC survivors?

### 2.3. Search strategy

A comprehensive literature search was performed across eleven databases, including Web of Science, PubMed, Cochrane Library, Embase, CINAHL, ProQuest, PsycINFO, ScienceDirect, CNKI, Wanfang Data Knowledge Service Platform, and VIP. The search covered the period from the inception of each database to August 20, 2025. Complete Chinese/English search strategies are available in the S2 File.

### 2.4. Inclusion and exclusion criteria

The inclusion criteria were developed using the PCC framework [19]. We included studies that involved adult participants (≥18 years) diagnosed with HNC (originating from the sinus, nasopharynx, oro and hypopharynx, larynx, oral cavity and salivary glands) who had completed all primary treatments. The studies needed to investigate the unmet supportive care needs of HNC survivors and observe these unmet needs in real-world settings such as community or home environments. We excluded studies where the full text was unavailable, as well as non-research publications including news reports, guidelines, reviews, and conference abstracts. Additionally, we excluded duplicate publications, retracted articles, and publications in languages other than Chinese or English.

### 2.5. Data abstract

**Fig 1:**
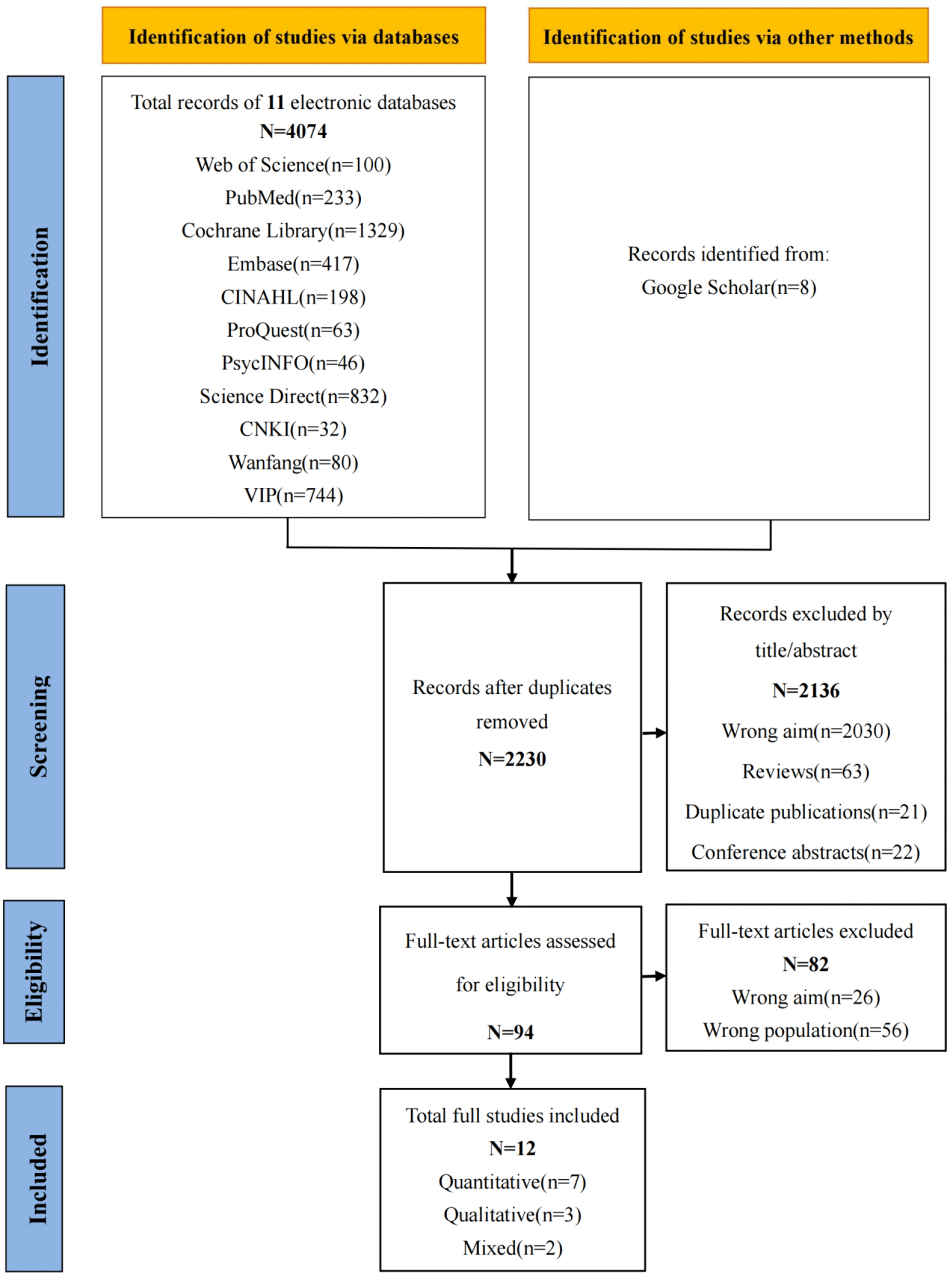
PRISMA flowchart of the literature search and selection process.

All retrieved records were imported into Zotero, and duplicates were removed. Subsequently, two independent researchers trained in evidence-based methodology screened titles and abstracts according to the inclusion criteria. Full texts of potentially eligible studies were then assessed. Disagreements were resolved through discussion with a third reviewer. Extracted data included: first author and publication year, country/region, assessment instrument, study type, sample size, domains of unmet supportive care needs, and factors associated with these needs. (Fig 1)

### 2.6. Data synthesis

For studies selected for full-text assessment, two reviewers independently evaluated each article to extract reported findings on unmet supportive care needs. To ensure a consistent synthesis across studies that used heterogeneous tools, we categorized all needs using the pre-existing framework of the Supportive Care Needs Survey 34-item Short-Form (SCNS-SF34) [20] and its HNC-specific module (SCNS-HNC) [21]. This framework encompasses the five SCNS-SF34 domains (psychological, health system and information, patient care and support, physical and daily living, and sexuality) and the two SCNS – HNC domains (HNC-specific functioning needs and HNC-specific life needs). Any disagreements between reviewers were resolved through consensus discussion.

## 3. Results

A total of 4074 articles were retrieved. After removing 1844 duplicates, the remaining 2230 unique records underwent title and abstract screening. After title and abstract screening, 2,136 records were excluded, primarily due to wrong aim (n = 2,030), reviews (n = 63), duplicate publications (n = 21), and conference abstracts (n = 22). We assessed 94 full-text articles, of which 82 were excluded, mainly because of wrong population (n = 56) and wrong aim (n = 26). 12 studies ultimately met all inclusion criteria and were included in the synthesis. Included studies originated from six developed regions: the United Kingdom (UK; n = 3) [22–24], the United States (USA; n = 3) [25–27], Netherlands (n = 3) [23,28,29], Switzerland (n = 1) [30], Hong Kong, China (n = 1) [31], and Taiwan, China (n = 1) [32]. Quantitative designs (n = 7) [23–25,27,28,30,32] predominated, while qualitative (n = 3) [26,29,33] and mixed-methods (n = 2) [22,31] approaches comprising the remainder. Sample sizes ranged from 31 to 483. Characteristics of the included studies are summarized in Table 1.

**Table 1:** Characteristics of included studies.

First author and year of publication	Country/region	Type of cancer	Assessment instrument	Study type	Sample size
D Molenaar [23] 2025	Netherlands	Oral cavity, Oropharynx, Hypopharynx, Larynx, Unknown primary	SCNS-SF34 [20], SCNS-HNC [21]	Quantitative	483
Ava Lorenc [22] 2022	UK	Unclear	–	Mixed	144
Cecilia Kim [25] 2021	USA	Unclear	A self-administered questionnaire	Quantitative	200
Gerben van Hinte [28] 2021	Netherlands	Oral cavity, Nasopharynx, Oropharynx, Larynx, Other	SCNS-SF34 [20], SCNS-HNC [21]	Quantitative	50
Sylvia L. Crowder [26] 2021	USA	Oral cavity, Oropharynx, Hypopharynx, Nasopharynx, Larynx	–	Qualitative	31
Arta Hoesseini [29] 2020	Netherlands	Larynx, Hypopharynx, Oral cavity, Oropharynx, Unknown primary	–	Qualitative	40
Winnie K.W. So [31] 2019	Hong Kong, China	Pharynx, Larynx, Tongue, Salivary gland	SCNS-SF34-C [34]	Mixed	338
Simon Andreas Mueller [30] 2019	Switzerland	Oral cavity, Larynx, Oropharynx, Hypopharynx, Nasopharynx, Nose and paranasal sinus, Unknown primary, Multiple	–	Quantitative	101
Morgan S Lee [27] 2016	USA	Oral cavity	SUNS [35]	Quantitative	342
Amy E. Richardson [33] 2015	UK	Unclear	–	Qualitative	83
M. WELLS [24] 2015	UK	Unclear	the Patient Concerns Inventory (PCI)-Concerns [36]	Quantitative	280
Chen SC [32] 2014	Taiwan, China	Oral cavity	Partners and caregivers supportive care needs survey [37]	Quantitative	102

### 3.1. Unmet supportive care needs

**Fig 2:**
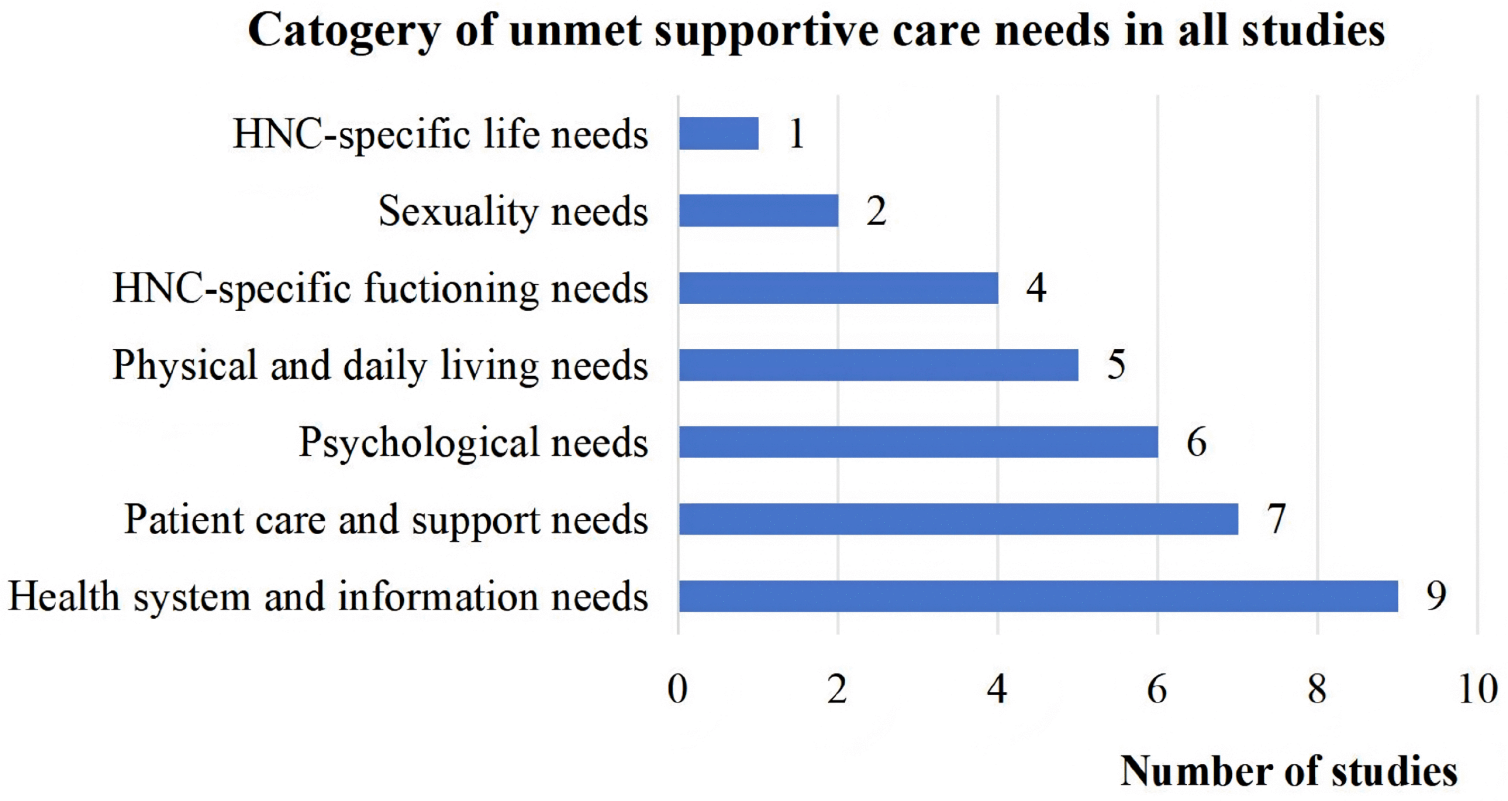
Total number of unmet supportive care needs categories in all studies.

HNC survivors reported unmet supportive care needs across health system/information, psychological, patient care/support, physical/daily living, sexuality, HNC-specific functioning and HNC-specific life domains. Detailed findings regarding these unmet supportive care needs were presented in Table 2. Based on the synthesis of the 12 included studies, the most frequently reported unmet need domains were health system/information (reported in 9 studies), patient care/support (7 studies), psychological needs (6 studies). (Fig 2)

**Table 2:** Unmet supportive care needs in HNC survivors.

Unmet supportive care needs	Articles
**Health system and information needs**	
Information about cancer which is under control or diminishing	[23,31]
Test results as soon as feasible	[23,31]
The benefits and side-effects of treatments	[23,25,32,33]
Treatment and care planning	[23,26]
Things that can help people with HNC get well	[23,25,31,32]
Being given explanations of those tests	[23]
More choice about which cancer specialist they see	[23,32]
Quick, direct, reliable and easy access to a clinician and expert advice	[22]
Being taken seriously and appropriate, timely responses, and feeling supported	[22]
Opportunities to discuss your concerns with the doctors	[32]
Adequate pain control	[32]
Information about recurrence	[22]
Information about prognosis	[29,32]
Multidisciplinary management	[26]
Regular follow-up service	[30]
One-on-one service	[33]
**Psychological needs**	
Uncertainty about the future	[23,27]
Concerns about the worries of those close to them	[23]
Feelings about death and dying	[23]
Fears about the cancer spreading or recurrence	[23,24,31,32]
Down or depressed or sadness	[23]
Anxiety and/or stressed or tired	[23,24,27]
Changes in physical ability	[27]
Bad memory or lack of focus	[27]
Understanding the experience of the person with cancer	[32]
Peer support	[22]
**Patient care and support needs**	
Having 1 hospital staff who you can talk to about condition, treatment and follow-up	[23,31]
Being treated like a person not just another case	[23]
Support for family and friends	[22]
Financial support or government benefits	[27,32]
Enough social support	[23,31,33]
**Physical and daily living needs**	
Lack of energy/tiredness	[23]
Pain	[24,28]
Work around the home	[28]
Not being able to do things you used to do	[28]
Monetary allowance for travel, treatment, and equipment expenses	[31]
Sleeping	[24]
Appearance	[24]
Eating problems	[24]
Appetite	[24]
Caring for patients on a practical level	[32]
**Sexuality needs**	
Information about sexual relationships	[23,33]
Changes in sexual feelings or their sexual relationships	[23]
**HNC-specific functioning needs**	
Dry mouth and/or sticky saliva	[23,24]
Mobility of neck or shoulders	[23,26,28]
Chewing and or swallowing	[23,24,26,28]
Hearing	[23,24]
Weight (underweight or overweight)	[23]
To be informed on nutrition	[23,26]
Speech/voice/being understood	[23,24]
Dental health/teeth	[24,26]
Taste change or loss	[24]
**HNC-specific life needs**	
Quit smoking	[23]
Quit drinking	[23]

#### 3.1.1. Health system and information needs.

Patient-reported unmet needs predominantly involved health and information domains (9 studies [22,23,25,26,29,31–33]). HNC survivors expressed the need for designated hospital staff to provide comprehensive consultations regarding, their condition, treatment, and follow-up [23,31],enhancing perceived support [22]. Access to physicians for addressing concerns was also essential [32]. Substantial unmet information needs persisted across the disease trajectory, including the important aspects of their care [23], aspects of managing illness and side-effects at home [23], treatment complications [25], treatment and care planning [26,31,32], recovery expectation [23,25,31,32]. Inadequate disease knowledge consistently exacerbated disease-related fears [22]. Providing timely and appropriate personalized prognosis assessments is beneficial [29]. Several unmet needs related to health system and information were not captured by the assessment tools, including information about recurrence [22], information about prognosis [29,32], multidisciplinary management [26], regular follow-up service [30], and one-on-one service [33].

#### 3.1.2. Patient care and support needs.

7 studies [22,23,26,27,31–33] reported on the patient care and support needs of HNC survivors. The identified unmet needs encompassed financial pressures [27,32], support for families and friends [22], being treated as an individual rather than just a case [23], and having one member of hospital staff with whom they can talk to about all aspects of the condition, treatment and follow-up [23]. Among the unmet patient care and support needs identified, financial pressures were specifically mentioned in 2 studies [27,32]. Support for family and friends [22] were also consistently highlighted as crucial. Furthermore, the degree of personalized care and the quality of communication emerged as significant areas of concern. Enough social support [23,31,33] related to patient care and supports were not identified by the assessment tools.

#### 3.1.3. Psychological needs.

Psychological needs represented another prominent area of unmet needs, identified in 6 studies [22–24,27,31,32]. These needs include fear of relapse [24,32] or cancer spreading [23,31],treatment-related fatigue [24,27], lack of confidence in life [27], uncertainty about the future [23,27], feeling of being misunderstood [32], concerns about the worries of those close to them [23], learning to feel in control of one’s situation [23], feelings about death and dying [23]. HNC survivors preferred one-on-one psychotherapy [33]. Within 6 months post-treatment, empathy from family and friends constituted the most valued psychological support [33]. Furthermore, mental health support proved essential for encouraging active engagement in follow-up care [33]. The assessment tools failed to capture peer support [22] related to psychological needs.

#### 3.1.4. Physical and daily living needs.

5 studies [23,24,28,31,32] reported significant unmet physical and daily living needs among HNC survivors. Fatigue [23,28] and pain [24,28] were the most frequently reported issues, substantially impairing daily functioning. Specific impacts included work interruption [28] and limitations in basic activities of daily living, including eating and sleeping [24]. Notably, HNC survivors clearly expressed an urgent need for practical caregiving support such as with bathing, changing dressings, or giving medications [32]. Financial concerns regarding travel, treatment and equipment costs were also identified [31].

#### 3.1.5. HNC-specific functioning and life needs.

4 studies [23,24,26,28] of the 12 included studies mentioned HNC-specific functional needs and 1 study [23] mentioned HNC-specific life needs. Unmet HNC-specific functional needs in HNC survivors included the management of symptoms such as dry mouth and/or sticky saliva, chewing and/or swallowing problems, mobility of neck or shoulders, hearing problems, speaking problems, taste change. Additionally, HNC survivors expressed a need for support in modifying lifestyle behaviors, such as smoking and alcohol cessation [23].

#### 3.1.6. Sexuality needs.

2 studies [23,31] identified unmet sexual needs among HNC survivors, including changes in their sexual relationships or sexual feelings [23], information about sexual relationships [23,31]. The most frequently cited unmet need was inadequate information concerning sexual relationships [23,31]. In addition, with regard to sexual desire, studies have shown that sexual desire persists strongly for 6 months to 2 years after treatment for HNC, with no significant decline observed during this period [23].

### 3.2. Unmet supportive care needs assessment tools

The unmet supportive care needs assessment tools employed in the included studies comprised the SCNS-SF34 [20], SCNS-SF34-C [38], SCNS-HNC [21], SUNS [39], The Patient Concerns Inventory (PCI)-Concerns [40], Partners and caregivers supportive care needs survey [41] and Self-administered questionnaire [25]. The SCNS-SF34 [20] and SCNS-HNC [21] were the most commonly used instruments, each employed in 2 studies. The domains covered by each assessment tool are detailed in Table 3.

**Table 3:** Domains of unmet supportive care needs assessment tool.

Unmet supportive care needs assessment tools	Health system and information needs	Psychological needs	Patient care and support needs	Physical and daily living needs	Sexuality needs	HNC-specific	
						Functioning needs	Life needs
SCNS-SF34 [20]	**√**	**√**	**√**	**√**	**√**	×	×
SCNS-SF34-C [38]	**√**	**√**	**√**	**√**	**√**	×	×
SCNS – HNC [21]	×	×	×	×	×	**√**	**√**
SUNS [39]	**√**	**√**	**√**	**√**	**√**	×	×
PCI – Concerns [40]	×	**√**	×	**√**	×	**√**	×
Partners and caregivers supportive care needs survey [41]	**√**	**√**	**√**	**√**	×	×	×
Self-administered questionnaire [25]	**√**	×	×	×	×	×	×

**√** Incorporate an evaluation of this domain, × Not incorporate an evaluation of this domain.

Among the 7 assessment tools identified, SCNS-SF34 [20], SCNS-SF34-C [38] and SUNS [39] demonstrated the greatest comprehensiveness, encompassing domains of health system/information, psychological, patient care/support, physical/daily living, and sexuality. However, these generic tools did not include modules for HNC-specific functional and lifestyle needs. SCNS-HNC [21], a tool designed specifically for HNC survivors, assesses both HNC-specific functional deficits and lifestyle needs.

### 3.3. Factors associated with unmet supportive care needs

#### 3.3.1. Factors associated with unmet health system and information needs.

In demographic factors, an annual income below $60,000 [27] was associated with unmet health system and information needs. In clinical factors, fewer years since diagnosis [27], recurrence of disease [30], occurrence of second primaries [30], and feeding tube usage [23] were associated with more unmet health system and information needs. In psychological factors, lower levels of role functioning [23], emotional functioning [23], neuroticism [23], fear of recurrence [23], and extraversion [23] were associated with unmet needs within health system and information.

#### 3.3.2. Factors associated with unmet patient care and support needs.

In demographic factors, female [27], younger age [27], non-white racial status [27], unmarried status [27] and disability [27] were associated with unmet needs across patient care and support needs. In clinical factors, fewer years since diagnosis [27] was associated with unmet patient care and support needs. In psychological factors, lower levels of role functioning [23], emotional functioning [23], neuroticism ^[^1^]^, lower physical/mental health status [27], extraversion [23], and fear of recurrence [23] were associated with unmet needs within patient care and support.

#### 3.3.3. Factors associated with unmet psychological needs.

In demographic factors, female [27], younger age [27], non-white racial status [27], unmarried status [27], Hispanic ethnicity [27], disability [27], and an annual income below $60,000 [27] were associated with unmet needs across psychological needs. HNC survivors experiencing weight gain [23] reported lower psychological needs. In clinical factors, fewer years since diagnosis [27] and stage 3 disease [23] were associated with unmet psychological needs. In psychological factors, lower levels of social functioning [23], neuroticism [23], anxiety [23], lower physical/mental health status [27] and fear of recurrence [23] were associated with unmet psychological needs.

#### 3.3.4. Factors associated with unmet physical and daily living needs.

In clinical factors, feeding tube usage [23] and problems with lateral flexion neck left [28] were associated with unmet physical and daily living needs. In psychological factors, lower levels of role functioning [23], higher levels of fatigue [23], higher pain [23], anxiety [23] were associated with physical and daily living needs.

#### 3.3.5. Factors associated with unmet sexuality needs.

Female survivors [23], painkillers usage [23], lower levels of social functioning [23], and anxiety [23] were associated with unmet sexuality needs.

#### 3.3.6. Factors associated with unmet HNC–specific needs.

Painkillers usage [23], higher levels of appetite loss [23], less constipation [23], swallowing problems [23], less sexuality [23], openness [23] and seeking social support [23] were associated with unmet HNC-specific functioning needs. Higher levels of appetite loss [23], less constipation [23], swallowing problems [23], less sexuality [23], lower levels of emotional functioning [23], trouble with social contact [23], problems with teeth [23], lower level on dry mouth [23] and higher level of coughing [23] were associated with unmet HNC-specific life needs.

## 4. Discussion

In our scoping review, we found that the three most frequently intertwined unmet supportive care needs were health/ information needs [22,23,25,26,29–33], patient care/support needs [22,23,26,27,31–33] and psychological needs [22–24,27,31,32]. These unmet needs collectively exert a profound adverse impact on HNC survivors’ quality of life. HNC survivors frequently report insufficient information [28], particularly in the areas of treatment effect, prognosis, and complication management. This may be attributed to the patient populations involved in the included studies generally possessed higher health literacy and socioeconomic status, consequently demonstrated a stronger motivation for self-management. When this proactive intention is not met through effective and accessible channels, the resulting information gap can further exacerbate feelings of anxiety and helplessness [22]. Therefore, unmet information needs extend beyond the simple transfer of knowledge; they represent a core clinical issue that directly impacts patients’ psychological adjustment and quality of life. Furthermore, the management of treatment-related complications is essential. These complications directly lead to severe restrictions in eating, communication, and daily activities [29], impair patients’ most fundamental physiological and social functions. These complex and intertwined functional problems may benefit from coordinated intervention by a multidisciplinary team (MDT). The MDT could extend beyond HNC specialists and psychologists to include pivotal allied health professionals. This includes nurse navigators, who coordinate care and guide patients through the complex healthcare system; speech-language pathologists, who are critical for rehabilitating swallowing and communication functions; dietitians, who manage malnutrition and support healing; and physiotherapists, who address impairments in mobility and strength. Future research should explore the feasibility and effectiveness of such comprehensive team-based approaches in addressing the multifaceted needs of HNC survivors. Additionally, telehealth solutions, such as online psychological counseling and remote functional training/guidance, can address some unmet needs. A study demonstrated that remote system monitoring and/or self-management via mobile apps was feasible for most HNC survivors [34]. Therefore, advancing telehealth applications for HNC survivors, combined with interdisciplinary team support, is essential to meet their multifaceted needs. Future research should investigate how telehealth can be optimally integrated with interdisciplinary support to meet the diverse needs of this population.

Current assessment tools fail to comprehensively capture the complex needs of HNC survivors. Several instruments exhibit structural gaps in critical domains. the PCI [40] lacks coverage of health system and information needs, sexuality concerns, and HNC-specific issues. Such gaps increase the risk of overlooking important unmet needs among survivors. Additionally, integrated approaches that combine general cancer needs assessments with HNC-specific scales remain substantially underutilized in clinical practice. This limitation leads to inadequate representation of HNC-specific needs, regarding swallowing difficulties and speech impairments, during routine evaluations. Furthermore, qualitative [26,29] and mixed studies [22] have identified several significant unmet needs not captured by existing standardized tools. These include needs for peer support, detailed information about prognosis and recurrence, and accessible MDT care. Although these qualitative studies typically involve smaller sample sizes, they reveal crucial unmet supportive care content that current assessments fail to address. Existing assessment tools may not adequately align with the complex realities of the growing HNC survivor population in terms of both content coverage and clinical relevance. Future research should consider the development of a comprehensive assessment tool that integrates both universal cancer indicators and HNC-specific dimensions, which could enable more accurate identification of survivors’ diverse supportive care needs.

Younger age [27], unmarried status [27], female gender [27], non-white racial status [27], advanced disease stage [23], significant treatment-related sequelae [23], and negative psychological characteristics [23] are associated with unmet supportive care needs among HNC survivors. Younger individuals often face unique challenges, including disruptions to their career and social identity, which compound the survivorship burden. A particularly noticeable gap exists in sexual health support for women, highlighting the inadequacy of current care models in addressing gender-specific issues. This underscores an urgent need for sensitive communication and intervention in this area. Notably, patients exhibiting negative psychological distress demonstrate a higher susceptibility to unmet needs across almost all domains. This indicates that psychological distress is not only a core supportive care need itself but also leading to higher level of unmet supportive care needs, consistent with other studies [35,42]. Increased attention and support for adverse psychological reactions in HNC survivors are warranted. Overall, sociodemographic differences, symptom burden, and psychological distress are associated with unmet supportive care needs in HNC survivors. Future clinical practice should focus on identifying high-risk individuals and providing personalized interventions, thereby transitioning from universal care to precision support. This approach will ultimately enhance the quality of life for HNC survivors.

This research provided a categorization and integration of unmet supportive care needs among HNC survivors. Significant unmet needs were identified across seven domains, including health system and information, psychological, patient care and support, physical and daily living, sexuality, HNC-specific functioning, and life needs. Critically, health system/information needs, psychological needs, and patient care/support needs emerged as the most prevalently reported. However, existing assessment instruments frequently lacked comprehensive coverage of HNC-specific dimensions. These gaps may obscure key clinical issues. There are significant interactions between unmet needs and demographic, clinical, and psychological factors, underscoring the necessity for individualized interventions.

## 5. Limitations

Several limitations warrant acknowledgment. First, caution is required when generalizing findings to developing countries due to fundamental disparities in healthcare systems, as the extant literature predominantly derives from developed nations. Second, all studies are from high-income regions in North American, European and Asia. Cultural elements from other high-income, low- and middle-income regions are not included, which may be associated with unmet social needs. Third, the predominance of cross-sectional designs impedes exploration of optimal assessment timing and fails to capture longitudinal dynamics of unmet needs across the disease trajectory. Fourth, while we compared the content coverage of unmet supportive care needs assessment tools, we did not assess validity, reliability, or measurement quality of these tools. Finally, the absence of formal literature quality appraisal necessitates consideration of the methodological rigor within included studies.

## 6. Conclusion

HNC survivors experience multiple unmet supportive care needs. Health system/information needs, patient care/support needs, and psychological needs were the most reported areas. HNC-specific requirements have not been fully explored. In addition, these needs of HNC survivors are associated with demographics, clinical, and other factors. These findings indicate that the unmet supportive care needs of HNC survivors are multidimensional and heterogeneous. There is a need to develop multidisciplinary care teams for HNC survivors in future. Furthermore, comprehensive assessment tools should be developed to fully understand the current landscape of unmet supportive care needs among HNC survivors.

## Supporting information

S1 File PRISMA checklist. (DOCX)

S2 File Search strategy. (DOCX)
