# Supplementary material for: Unmet supportive care needs of head and neck cancer survivors: A scoping review
Source: PLoS One. 2026 Apr 15;21(4):e0347295. doi: 10.1371/journal.pone.0347295 (PMC13082597; doi:10.1371/journal.pone.0347295)
Supplement: S2 File — (DOCX) [file pone.0347295.s002.docx]

| Name of the database | | ***PubMed (to 2025-08-20)*** | |
| --- | --- | --- | --- |
| Dates of the search | | 2025.8.20 | |
|  | **Search terms** | | **Number of hits** |
| 1 | ("Head and Neck Neoplasms"[Mesh]) | | 374,791 |
| 2 | (Head[Title/Abstract] OR Neck Neoplasm[Title/Abstract] OR Hea  d, Neck Neoplasms[Title/Abstract] OR Neoplasms, Head[Title/Ab  stract] OR Neck[Title/Abstract] OR Head Neoplasms[Title/Abstra  ct] OR Head Neoplasm[Title/Abstract] OR Neoplasm, Head[Title/  Abstract] OR Neoplasms, Head[Title/Abstract] OR Neck Neoplas  ms[Title/Abstract] OR Neck Neoplasm[Title/Abstract] OR Neopla  sm, Neck[Title/Abstract] OR Neoplasms, Neck[Title/Abstract] OR  Cancer of Head[Title/Abstract] AND Neck[Title/Abstract] OR C  ancer of the Head[Title/Abstract] OR Neck[Title/Abstract] OR H  ead[Title/Abstract] AND Neck Cancer[Title/Abstract] OR Upper  Aerodigestive Tract Neoplasms[Title/Abstract] OR Upper Aerodig  estive Tract Neoplasm[Title/Abstract] OR UADT Neoplasm[Title/  Abstract] OR Neoplasms, UADT[Title/Abstract] OR Neoplasm,  UADT[Title/Abstract] OR Neoplasms, Upper Aerodigestive Tract  [Title/Abstract] OR UADT Neoplasms[Title/Abstract] OR Cancer  of Neck[Title/Abstract] OR Neck Cancers[Title/Abstract] OR Ca ncer of the Neck[Title/Abstract] OR Neck Cancer[Title/Abstract] OR Cancer, Neck[Title/Abstract] OR Cancers, Neck[Title/Abstrac t] OR Cancer of Head[Title/Abstract] OR Head Cancers[Title/Ab stract] OR Cancer of the Head[Title/Abstract] OR Head Cancer [Title/Abstract] OR Cancer, Head[Title/Abstract] OR Cancers, He ad[Title/Abstract]) | | 45,491 |
| 3 | #1 OR #2 | | 387,796 |
| 4 | (((((((supportive care need[Title/Abstract]) OR (supportive care n eeds[Title/Abstract])) OR (care needs[Title/Abstract])) OR (care n | | 48,879 |

|  | eed[Title/Abstract])) OR (unmet needs[Title/Abstract])) OR (unme t need[Title/Abstract])) OR (SCNs[Title/Abstract])) |  |
| --- | --- | --- |
| 5 | #3 AND #4 | 233 |
| Filters：Full text, English, from establishment - 2025/08/20 | | |

| Name of the database | | ***CNKI (to 2025-08-20)*** | |
| --- | --- | --- | --- |
| Dates of the search | | 2025.8.20 | |
|  | **Search terms** | | **Number of hits** |
| 1 | (主题=头颈癌 + 头颈癌肿瘤 + 鼻咽癌 + 鼻咽肿瘤 + 口咽癌 + 口咽肿瘤) | | 36072 |
| 2 | (主题=支持性照护需求 + 支持性照护 + 照护需求 + 未满足的需求 +护理需求 + 支持性照护需求框架) | | 9163 |
| 3 | #1 AND #2 | | 32 |
| Filters: from establishment - 2025/08/20 | | | |
