# Supplementary material for: Unmet supportive care needs of head and neck cancer survivors: A scoping review
Source: PLoS One. 2026 Apr 15;21(4):e0347295. doi: 10.1371/journal.pone.0347295 (PMC13082597; doi:10.1371/journal.pone.0347295)
Supplement: Attachment — Submitted filename: Response to Reviewers.docx [file pone.0347295.s004.docx]

Dear Editor,

Thank you very much for your letter and editor's positive feedback on our manuscript entitled “Unmet supportive care needs of head and neck cancer survivors: A scoping review” (Manuscript ID: PONE-D-25-46612). We appreciate the opportunity to revise the manuscript and address editor's comments. According to editor's request, we have carefully revised the manuscript and point-by-point addressed the academic editor and reviewers’ concerns. **All revisions made in ‘Revised Manuscript with Track Changes’ have been highlighted in yellow and annotated in the revised manuscript**. We hope that our manuscript could be considered for possible acceptance for publication in PLOS ONE. The main corrections in the manuscript and the responds to the editor's comments are as follows:

**Reviewer #1:**

1. - A major part of the study includes evaluation of existing tools to screen unmet needs. This could be clarified in the abstract.

**Response:** We sincerely thank the reviewer#1 for this critical insight. The aim in the revised abstract has included the evaluation of existing tools to screen unmet needs. This change is reflected in the revised manuscript on **line 2, page 2.**

2. - Given the variety of studies (quantitative, qualitative, and mixed methodologies), could the authors comment and include whether any of the qualitative or mixed studies found any domains of supportive needs that were not identified in the evaluation tools?

**Response:** We thank the reviewer#1 for this valuable suggestion. The Results and discussion sections now include findings on the domains of supportive needs found through qualitative and mixed-methods research, which were not identified by the evaluation tools. The change is reflected in the revised manuscript on **line116-119/127-128/137, page 10 and line 240-244, page 15.**

3. - I found Table 3 is thorough though a bit hard to digest as a reader. Would consider consolidating the prose or reformatting to consolidate the table.

**Response:** We sincerely thank the reviewer#1 for this valuable feedback. Table 3 has been consolidated and reformatted to improve readability and is now presented as **Table 2**. Please see the changes in the revised manuscript on **line 105-107, page 8-9.**

4. - In the limitations, would also consider including that cultural aspects are not included in these studies extracted. All studies are from high-income North American and European countries. Cultural elements from other high-income countries include from other LMICs are not included and may influence unmet social needs.

**Response:** We thank the reviewer#1 for raising this critical point. We have added a point about cultural aspects to the limitations section. Please see the changes in the revised manuscript on **line 276-278, page 17**.

**Reviewer #2:**

Introduction

1. In the first sentence of the article, the authors mention that head and neck cancers are the 6th most common type of malignancy. However, this epidemiologic definition usually only includes cancers of the mucosal lining including sinus, nasopharynx, oral and hypopharynx, larynx, oral cavity and sometimes salivary glands. Cutaneous malignancies and thyroid cancers are excluded from this definition.

**Response:** We thank the reviewer#2 for this critical clarification. The first sentence of the introduction has been revised to align with the standard epidemiological definition. The text now clearly states that our review focuses on cancers of the sinus, nasopharynx, oro and hypopharynx, larynx, oral cavity and salivary glands. Please see the changes in the revised manuscript on **line 18-19, page 3.**

2. From line 27-37, the authors describe what type of complications patients may experience and I have several issues with how this is phrased:

- The sentence “While life-saving, treatments frequently incur substantial adverse effects that generate persistent multidimensional burdens for HNC survivors throughout post-treatment survivorship” is extremely vague and lacks direction. I would suggest a broad description of how patients are affected “head and neck cancer treatment will have a significant impact on patients’ appearances as well as breathing & eating function […].

- Some specific side effects of treatment are mentioned with the exclusion of surgical treatment. I would advise that the authors mention all modalities of treatment with their main side effects without being too granular. The focus should be on radiation therapy and surgery as these are the modalities with the most impact.

**Response:** -We thank the reviewer#2 for this suggestion. The vague sentence has been replaced with a more direct description of the impact on appearance, breathing, and eating, as recommended. Please see the changes in the revised manuscript on **line 27-30, page 3.**

-We thank the reviewer#2 for this valuable suggestion. The relevant paragraph has been revised to mention all treatment modalities and their main side effects (without being too granular), with primary emphasis on radiation therapy and surgery. Please see the changes in the revised manuscript on **line 30-34, page 3.**

Review questions

3. I would like the first question to be “what are the unmet needs” and the second question to be “what tools are used.” This order makes it more like “what do we know so far” and “how is it usually measured/are we missing anything”

**Response:** We thank the reviewer#2 for this suggestion. The review questions have been reordered accordingly, addressing "what are the unmet needs" first, followed by "what tools are used.". Please see the changes in the revised manuscript on **line 54-55, page 4.**

Inclusion and exclusion criteria

4. I believe inclusion and exclusion criteria should be written as sentences and not point form “we included…” “we excluded …”

**Response:** We thank the reviewer#2 for this suggestion. The inclusion and exclusion criteria have been reformatted from bullet points into a narrative paragraph. Please see the updated text on **line 63-70, page 5.**

5. For the scoping review to be coherent in terms of patient population, I would include only aerodigestive tract cancers (as mentioned in my first comment). Cutaneous and thyroid malignancies should be excluded from the review as they have completely different prognoses and treatment profiles.

**Response:** We thank the reviewer#2 for reinforcing this critical point. The inclusion criteria have been revised to specify that only patients with aerodigestive tract cancers (**originating from the sinus, nasopharynx, oro and hypopharynx, larynx, oral cavity and salivary glands**) were included, while those with cutaneous or thyroid malignancies were excluded. This change has been made in the methods section on **line 64-65, page 5.**

Data synthesis

6. This paragraph was a bit unclear to me. Studies presented their own tool, but your reclassified according to the SCNS-SF34? This section needs to be clarified.

**Response:** We sincerely thank the reviewer#2 for raising this point. The paragraph has been revised to clarify that we applied the **pre-existing** frameworks of the SCNS-SF34 and SCNS-HNC to synthesize and categorize the unmet needs reported across studies. This change has been made in the methods section on **line 80-87, page 6.**

Results

7. The types of cancers in each study should be mentioned in table 1.

**Response:** We thank the reviewer#2 for this suggestion. The types of HNC for each included study have now been added to Table 1. This change has been made on **line 96, page 7.**

8. Again, as for the review questions, I would order the actual unmet needs results before what tools were used.

**Response:** We thank the reviewer#2 for this suggestion. The results have been reordered to present the unmet needs before the description of the assessment tools. The change has been made in the methods section on **line 98-170, page 8-13.**

9. Line 123-124: Health system/information, psychological, patient care/support needs were the top 3 unmet needs. **These were the results you find in your review, it does not mean it is the truth.

**Response:** We thank the reviewer#2 for this comment. The sentence has been revised to clarify that the identified top 3 unmet needs are **based on the synthesis of the included studies.** Please see the updated sentence on **line 101-103, page 8.**

10. I think table 4 should be removed and kept as the paragraph describing what are the main factors that were found to influence unmet supportive care needs. The table is a bit confusing as is.

**Response:** We thank the reviewer#2 for this suggestion. As recommended, we have removed Table 4 and integrated some paragraphs describing the main factors that were found to influence unmet supportive care needs. Please see the updated sentence on **line 171-208, page 13-14.**

11. At line 220, there is a mention of thyroid cancer patients. I strongly believe this cancer population should not be included in the study.

**Response:** We thank the reviewer#2 for this insightful comment. We have now systematically removed all mentions of thyroid cancer patients throughout the manuscript to ensure consistency with our focused scope on HNC. Please see the updated sentence on **line 188-189, page 13.**

Discussion

12. Lines 242-246: these lines should be removed, no need to justify the study at the start of the discussion. A discussion should start with what the study was and what the main finding was. “In our scoping review, we found that the 3 most frequently unmet supportive care needs were […]”

**Response:** We thank the reviewer#2 for this guidance. The opening paragraph of the discussion now directly presents the primary results, beginning with: "In our scoping review, we found that the 3 most frequently unmet supportive care needs were...". Please see the updated sentence on **line 210-212, page 14.**

13. Again, start by describing unmet needs and then go on with what tools were used.

**Response:** We thank the reviewer#2 for this suggestion. The sentences in the discussion have been reordered to first describe the unmet needs and then address the tools used. Please see the updated sentence on **line 210-249, page 14-16.**

14. Line 262: authors report a high willingness to pay and refer to a qualitative study that inherently has very few patients/patients willing to participate in research studies. This is likely a biased sample and I would refrain from mentioning this in the discussion. A significant portion of HNC patients come from a lower sociodemographics background and it would be hard for any head & neck oncologist to believe their patients have the funds/willingness to pay out of pocket for psychology services.

**Response:** We thank the reviewer#2 for this suggestion. The mention of high willingness to pay has been removed from the discussion. Please see the updated sentence on **line 210-233, page 14-15.**

15. Lines 274-279: I agree with the authors that multidisciplinarity helps with the care of patients and survivors. I would go further and mention the impact of nurse navigators and other allied health professionals (SLP, dietician, physiotherapists, etc) as part of the patients’ care teams.

**Response:** We sincerely thank the reviewer#2 for this suggestion. We have expanded the discussion to explicitly include the impact of nurse navigators and other allied health professionals such as speech-language pathologists, dietitians, and physiotherapists. Please see the updated sentence on **line 224-229, page 15.**

16. Overall comment on the discussion is to be careful not to repeat all the results – focus on few significant results and put them in perspective.

**Response:** We thank the reviewer#2 for this critical guidance. The discussion has been revised to focus on interpreting the key findings and their implications, rather than repeating the results. (**line 212-223/250-264, page 14-16**)

Conclusion

17. Lines 325-328: this sentence is too long and too vague. Focus on the key message of this scoping review. What do you want people to know? What is the main thing we should focus on/study after reading your scoping review?

**Response:** We thank the reviewer#2 for this constructive feedback. The conclusion has been revised to present a clearer and more focused key message, emphasizing the need for multidisciplinary care teams and comprehensive assessment tools. (**line 286-289, page 17**)

We tried our best to improve the manuscript and made some changes in the manuscript. These changes will not influence the primary content and framework of the manuscript. Furthermore, we confirm that the manuscript has been revised to meet PLOS ONE's style requirements using the provided templates. The Data Availability Statement has been updated to include the repository name. Captions for Supporting Information files have been added at the end of the manuscript, with all in-text citations updated accordingly.

We appreciate for editor's warm work earnestly, and hope that the correction will meet with approval. Once again, thank you very much for your comments and suggestions.

Sincerely yours,

Gu-Qing Zeng, Ph.D. Professor and Director
School of Nursing, Hengyang Medical School, University of South China, Hengyang 421001, P.R. China
Tel: 86-734-8281821
Fax: 86-734-8281344
E-mail: zengguqing0123@163.com
