# Supplementary material for: Unmet supportive care needs of head and neck cancer survivors: A scoping review
Source: PLoS One. 2026 Apr 15;21(4):e0347295. doi: 10.1371/journal.pone.0347295 (PMC13082597; doi:10.1371/journal.pone.0347295)
Supplement: Attachment — Submitted filename: Response_to_Reviewers_auresp_2.docx [file pone.0347295.s005.docx]

Dear Editor,

Thank you very much for your letter and editor's positive feedback on our manuscript entitled “Unmet supportive care needs of head and neck cancer survivors: A scoping review” (Manuscript ID: PONE-D-25-46612). We appreciate the opportunity to revise the manuscript and address editor's comments. According to editor's request, we have carefully revised the manuscript and point-by-point addressed the academic editor’s concerns. **All revisions made in ‘Revised Manuscript with Track Changes’ have been highlighted in yellow in the revised manuscript**. We hope that our manuscript could be considered for possible acceptance for publication in PLOS ONE. The main corrections in the manuscript and the responds to the editor's comments are as follows:

- Firstly, it is unclear why only 434 records were screened out of 2230 retrieved. Please update your PRISMA diagram to identify the 1796 papers that were removed before screening and indicate why these were removed.

**Response:** Thank you for your valuable feedback. We have carefully updated our PRISMA flow diagram in accordance with your comment. **The updated figure file has been uploaded to the submission system.**

- Secondly, please note that PLOS ONE does not copyedit accepted manuscripts and that one of our criteria for publication is that articles must be presented in an intelligible fashion and written in clear, correct, and unambiguous English (https://journals.plos.org/plosone/s/criteria-for-publication#loc-5). We suggest you have a fluent English-language speaker thoroughly copyedit your manuscript for language usage, spelling, and grammar. For example, there are a number of inappropriate uses of 'etc.' in various places (such as lines 4, 66, 134, 150, 242). A second example is that 'two reviewers' should be used in preference to '2 reviewers'. Please note that these examples are not exhaustive.

**Response:** Thank you for your valuable feedback on language quality. We have thoroughly revised the manuscript in response to your suggestions and have had the entire text copyedited by a fluent English-language speaker to ensure clarity and correctness. In addition, all specific content mentioned in your comments are available in **Line 3-4, 64, 79, 134, 147-149 and 240-241.**

Following the editorial guidance, we have carefully reviewed all comments. We confirm that no reviewer suggestions required citing additional works, and we have verified the completeness and accuracy of the reference list, including confirming that no retracted articles are cited.

We appreciate for editor's warm work earnestly, and hope that the correction will meet with approval. Once again, thank you very much for your comments and suggestions.

Sincerely yours,

Gu-Qing Zeng, Ph.D. Professor and Director
School of Nursing, Hengyang Medical School, University of South China, Hengyang 421001, P.R. China
Tel: 86-734-8281821
Fax: 86-734-8281344
E-mail: zengguqing0123@163.com
