# Supplementary material for: Unmet supportive care needs of head and neck cancer survivors: A scoping review
Source: PLoS One. 2026 Apr 15;21(4):e0347295. doi: 10.1371/journal.pone.0347295 (PMC13082597; doi:10.1371/journal.pone.0347295)
Supplement: Attachment — Submitted filename: Response_to_Reviewers_auresp_3.docx [file pone.0347295.s006.docx]

Dear Editor,

Thank you very much for your letter and editor's positive feedback on our manuscript entitled “Unmet supportive care needs of head and neck cancer survivors: A scoping review” (Manuscript ID: PONE-D-25-46612). We appreciate the opportunity to revise the manuscript and address reviewers' comments. According to editor's request, we have carefully revised the manuscript and point-by-point addressed the reviewers’ concerns. **All revisions made in ‘Revised Manuscript with Track Changes’ have been highlighted in yellow and annotated in the revised manuscript**. We hope that our manuscript could be considered for possible acceptance for publication in PLOS ONE. The main corrections in the manuscript are as follows:

**Reviewer #3:**

1. Study Selection Transparency

While a PRISMA flow diagram is referenced, the manuscript would benefit from clearer reporting of the study selection process. Specifically:

The number of duplicates removed should be explicitly stated.

Reasons for full-text exclusions should be categorized and quantified.

A brief textual summary of the most common exclusion reasons in the Results section would enhance clarity and reproducibility.

**Response:** We sincerely thank the reviewer#3 for this critical insight. We have revised the manuscript accordingly. These changes are reflected in the revised manuscript on **line 87-91, page 6.**

2. Clarification of “Top Three” Unmet Needs

The manuscript identifies health system/information, patient care/support, and psychological needs as the “top three” unmet needs. However, it is unclear how this ranking was determined. Please clarify whether this classification is based on:

Frequency of reporting across included studies,

Number of unmet items,

Proportion of participants reporting these needs,

Or another defined metric.

Without an explicit operational definition, the phrasing may overstate the strength of the evidence.

**Response:** We sincerely thank the reviewer#3 for this critical insight. We have revised the manuscript accordingly. This change is reflected in the revised manuscript on **line 102-104, page 8.**

3. Framing of Conclusions

As this is a scoping review, the primary aim is evidence mapping rather than causal inference or prescriptive recommendation. Some parts of the Discussion (e.g., recommendations regarding multidisciplinary team expansion and telehealth integration) should be more clearly framed as implications or future directions rather than conclusions directly derived from the data.

Additionally, the statement that this study provides the “first categorization and integration” of unmet needs among HNC survivors should be qualified unless clearly supported by the literature.

**Response:** We sincerely thank the reviewer#3 for this critical insight. We have revised the **Discussion section accordingly**. This change is reflected in the revised manuscript on **line 224-225/230-231/235-237/267-268, page 15.**

4. Assessment Tool Discussion

The comparison of supportive care needs assessment instruments is a valuable contribution. However, critiques regarding tool “incompleteness” are presented without formal psychometric evaluation. If assessment of validity, reliability, or measurement quality was beyond the scope of this review, this should be clearly acknowledged in the Methods and/or Limitations section.

**Response:** We sincerely thank the reviewer#3 for this critical insight. To address this concern, we have revised the **Limitations section.** This change is reflected in the revised manuscript on **line 282-283, page 17.**

5. Influencing Factors

The section discussing demographic, clinical, and psychological correlates is informative but could be better organized. Consider grouping findings by unmet need domain and clarifying whether reported associations were adjusted or unadjusted in the original studies. Causal language should be avoided unless supported by longitudinal evidence.

One statement regarding education level and psychological needs appears inconsistent and should be clarified.

**Response:** We sincerely thank the reviewer#3 for this critical insight. We have substantially revised this section to address your suggestions. Detailed changes can be found on **line 171-210, page 13.** Specifically, the revision about the statement regarding education level can be found on **line 189-192, page 13-14.**

6. Language and Presentation

The manuscript is generally written in clear academic English. Minor revisions are needed to:

Replace informal phrasing (e.g., “lots of unmet needs”)

Standardize terminology across need domains.

Correct spacing issues (e.g., “HNC-specific” formatting).

Improve conciseness in parts of the Introduction and Discussion.

No concerns regarding dual publication or research ethics are apparent.

**Response:** We sincerely thank the reviewer#3 for this critical insight. We have carefully revised the manuscript according to your suggestions. Specifically, informal phrasing (e.g., "lots of unmet needs") has been replaced with more formal academic expressions. These changes can be found on **line 13, page 2.**

We appreciate for editor's warm work earnestly, and hope that the correction will meet with approval. Once again, thank you very much for your comments and suggestions.

Sincerely yours,

Gu-Qing Zeng, Ph.D. Professor and Director
School of Nursing, Hengyang Medical School, University of South China, Hengyang 421001, P.R. China
Tel: 86-734-8281821
Fax: 86-734-8281344
E-mail: zengguqing0123@163.com
