# Supplementary material for: Unmet supportive care needs of head and neck cancer survivors: A scoping review
Source: PLoS One. 2026 Apr 15;21(4):e0347295. doi: 10.1371/journal.pone.0347295 (PMC13082597; doi:10.1371/journal.pone.0347295)
Supplement: Peer review history

# Peer review: Decision Letter 0

**DOI:** 10.1371/journal.pone.0347295.r001

**Authors:** Jen Edwards

21 Oct 2025

Dear Dr. Zeng,

Thank you for submitting your manuscript to PLOS ONE. After careful consideration, we feel that it has merit but does not fully meet PLOS ONE’s publication criteria as it currently stands. Therefore, we invite you to submit a revised version of the manuscript that addresses the points raised during the review process.

Please submit your revised manuscript by Dec 05 2025 11:59PM. If you will need more time than this to complete your revisions, please reply to this message or contact the journal office at plosone@plos.org. When you're ready to submit your revision, log on to https://www.editorialmanager.com/pone/ and select the 'Submissions Needing Revision' folder to locate your manuscript file.. When you're ready to submit your revision, log on to https://www.editorialmanager.com/pone/ and select the 'Submissions Needing Revision' folder to locate your manuscript file.. When you're ready to submit your revision, log on to https://www.editorialmanager.com/pone/ and select the 'Submissions Needing Revision' folder to locate your manuscript file.. When you're ready to submit your revision, log on to https://www.editorialmanager.com/pone/ and select the 'Submissions Needing Revision' folder to locate your manuscript file.

- A rebuttal letter that responds to each point raised by the academic editor and reviewer(s). You should upload this letter as a separate file labeled 'Response to Reviewers'.
- A marked-up copy of your manuscript that highlights changes made to the original version. You should upload this as a separate file labeled 'Revised Manuscript with Track Changes'.
- An unmarked version of your revised paper without tracked changes. You should upload this as a separate file labeled 'Manuscript'.

If you would like to make changes to your financial disclosure, please include your updated statement in your cover letter. Guidelines for resubmitting your figure files are available below the reviewer comments at the end of this letter.

If applicable, we recommend that you deposit your laboratory protocols in protocols.io to enhance the reproducibility of your results. Protocols.io assigns your protocol its own identifier (DOI) so that it can be cited independently in the future. For instructions see: https://journals.plos.org/plosone/s/submission-guidelines#loc-laboratory-protocols. Additionally, PLOS ONE offers an option for publishing peer-reviewed Lab Protocol articles, which describe protocols hosted on protocols.io. Read more information on sharing protocols at . Additionally, PLOS ONE offers an option for publishing peer-reviewed Lab Protocol articles, which describe protocols hosted on protocols.io. Read more information on sharing protocols at . Additionally, PLOS ONE offers an option for publishing peer-reviewed Lab Protocol articles, which describe protocols hosted on protocols.io. Read more information on sharing protocols at . Additionally, PLOS ONE offers an option for publishing peer-reviewed Lab Protocol articles, which describe protocols hosted on protocols.io. Read more information on sharing protocols at https://plos.org/protocols?utm_medium=editorial-email&utm_source=authorletters&utm_campaign=protocols....

We look forward to receiving your revised manuscript.

Kind regards,

Jen Edwards

Staff Editor

PLOS ONE

**Journal Requirements:**

When submitting your revision, we need you to address these additional requirements.

1. Please ensure that your manuscript meets PLOS ONE's style requirements, including those for file naming. The PLOS ONE style templates can be found at

https://journals.plos.org/plosone/s/file?id=wjVg/PLOSOne_formatting_sample_main_body.pdf and

https://journals.plos.org/plosone/s/file?id=ba62/PLOSOne_formatting_sample_title_authors_affiliations.pdf

2. Please note that your Data Availability Statement is currently missing the repository name. If your manuscript is accepted for publication, you will be asked to provide these details on a very short timeline. We therefore suggest that you provide this information now, though we will not hold up the peer review process if you are unable.

3. When completing the data availability statement of the submission form, you indicated that you will make your data available on acceptance. We strongly recommend all authors decide on a data sharing plan before acceptance, as the process can be lengthy and hold up publication timelines. Please note that, though access restrictions are acceptable now, your entire data will need to be made freely accessible if your manuscript is accepted for publication. This policy applies to all data except where public deposition would breach compliance with the protocol approved by your research ethics board. If you are unable to adhere to our open data policy, please kindly revise your statement to explain your reasoning and we will seek the editor's input on an exemption. Please be assured that, once you have provided your new statement, the assessment of your exemption will not hold up the peer review process.

4.  Please include captions for your Supporting Information files at the end of your manuscript, and update any in-text citations to match accordingly. Please see our Supporting Information guidelines for more information: http://journals.plos.org/plosone/s/supporting-information.

5. If the reviewer comments include a recommendation to cite specific previously published works, please review and evaluate these publications to determine whether they are relevant and should be cited. There is no requirement to cite these works unless the editor has indicated otherwise.

**Additional Editor Comments:**

The manuscript has been evaluated by two reviewers and their comments are available below.

The reviewers have raised a number of concerns that need attention. They request clarification of the text in a number of areas to improve readability and further discussion of study limitations and findings.

Could you please revise the manuscript to carefully address the concerns raised?

[Note: HTML markup is below. Please do not edit.]

Reviewers' comments:

Reviewer's Responses to Questions

**Comments to the Author**

1. Is the manuscript technically sound, and do the data support the conclusions?

Reviewer #1: Yes

Reviewer #2: Yes

**********

2. Has the statistical analysis been performed appropriately and rigorously?

Reviewer #1: N/A

Reviewer #2: Yes

**********

3. Have the authors made all data underlying the findings in their manuscript fully available?

The PLOS Data policy requires authors to make all data underlying the findings described in their manuscript fully available without restriction, with rare exception (please refer to the Data Availability Statement in the manuscript PDF file). The data should be provided as part of the manuscript or its supporting information, or deposited to a public repository. For example, in addition to summary statistics, the data points behind means, medians and variance measures should be available. If there are restrictions on publicly sharing data—e.g. participant privacy or use of data from a third party—those must be specified.requires authors to make all data underlying the findings described in their manuscript fully available without restriction, with rare exception (please refer to the Data Availability Statement in the manuscript PDF file). The data should be provided as part of the manuscript or its supporting information, or deposited to a public repository. For example, in addition to summary statistics, the data points behind means, medians and variance measures should be available. If there are restrictions on publicly sharing data—e.g. participant privacy or use of data from a third party—those must be specified.

Reviewer #1: Yes

Reviewer #2: Yes

**********

4. Is the manuscript presented in an intelligible fashion and written in standard English?

Reviewer #1: Yes

Reviewer #2: Yes

**********

Reviewer #1: Thank you to the authors for this manuscript. As the study highlights, head and neck cancer survivorship is complex given all the challenges that patients experience. The strengths of this study include the thorough search and range of studies the authors note. Specifically, they compare differences in evaluation tools, discuss the most common unmet supportive care needs, and propose areas of intervention including consolidating the strengths of screening tools and creation of multidisciplinary teams to address survivorship comprehensively. The authors also place the findings in context, noting that the studies are from all high-income countries.

Some suggestions below

- A major part of the study includes evaluation of existing tools to screen unmet needs. This could be clarified in the abstract.

- Given the variety of studies (quantitative, qualitative, and mixed methodologies), could the authors comment and include whether any of the qualitative or mixed studies found any domains of supportive needs that were not identified in the evaluation tools?

- I found Table 3 is thorough though a bit hard to digest as a reader. Would consider consolidating the prose or reformatting to consolidate the table.

- In the limitations, would also consider including that cultural aspects are not included in these studies extracted. All studies are from high-income North American and European countries. Cultural elements from other high-income countries include from other LMICs are not included and may influence unmet social needs.

Reviewer #2: I commend the authors for undertaking this important and timely work. Head and neck cancer survivors often experience significant disability following long and excruciating treatments, and improving their quality of care is of utmost importance. I generally found the article well written and informative; however, I recommend that substantial revisions be made before it is suitable for publication. Below is a point-by-point review of the manuscript.

Introduction

1. In the first sentence of the article, the authors mention that head and neck cancers are the 6th most common type of malignancy. However, this epidemiologic definition usually only includes cancers of the mucosal lining including sinus, nasopharynx, oro and hypopharynx, larynx, oral cavity and sometimes salivary glands. Cutaneous malignancies and thyroid cancers are excluded from this definition.

2. From line 27-37,the authors describe what type of complications patients may experience and I have several issues with how this is phrased:

- The sentence “While life-saving, treatments frequently incur substantial adverse effects that generate persistent multidimensional burdens for HNC survivors throughout post-treatment survivorship” is extremely vague and lacks direction. I would suggest a broad description of how patients are affected “head and neck cancer treatment will have a significant impact on patients’ appearances as well as breathing & eating function […].

- Some specific side effects of treatment are mentioned with the exclusion of surgical treatment. I would advise that the authors mention all modalities of treatment with their main side effects without being too granular. The focus should be on radiation therapy and surgery as these are the modalities with the most impact.

Review questions

3. I would like the first question to be “what are the unmet needs” and the second question to be “what tools are used.” This order makes it more like “what do we know so far” and “how is it usually measured/are we missing anything”

Inclusion and exclusion criteria

4. I believe inclusion and exclusion criteria should be written as sentences and not point form “we included…” “we excluded …”

5. For the scoping review to be coherent in terms of patient population, I would include only aerodigestive tract cancers (as mentioned in my first comment). Cutaneous and thyroid malignancies should be excluded from the review as they have completely different prognoses and treatment profiles.

Data synthesis

6. This paragraph was a bit unclear to me. Studies presented their own tool, but your reclassified according to the SCNS-SF34? This section needs to be clarified.

Results:

7. The types of cancers in each study should be mentioned in table 1.

8. Again, as for the review questions, I would order the actual unmet needs results before what tools were used.

9. Line 123-124: Health system/information, psychological, patient care/support needs were the top 3 unmet needs. **These were the results you find in your review, it does not mean it is the truth

10. I think table 4 should be removed and kept as the paragraph describing what are the main factors that were found to influence unmet supportive care needs. The table is a bit confusing as is.

11. At line 220, there is a mention of thyroid cancer patients. I strongly believe this cancer population should not be included in the study.

Discussion

12. Lines 242-246: these lines should be removed, no need to justify the study at the start of the discussion. A discussion should start with what the study was and what the main finding was. “In our scoping review, we found that the 3 most frequently unmet supportive care needs were […]”

13. Again, start by describing unmet needs and then go on with what tools were used.

14. Line 262: authors report a high willingness to pay and refer to a qualitative study that inherently has very few patients/patients willing to participate in research studies. This is likely a biased sample and I would refrain from mentioning this in the discussion. A significant portion of HNC patients come from a lower sociodemographics background and it would be hard for any head & neck oncologist to believe their patients have the funds/willingness to pay out of pocket for psychology services.

15. Lines 274-279: I agree with the authors that multidisciplinarity helps with the care of patients and survivors. I would go further and mention the impact of nurse navigators and other allied health professionals (SLP, dietician, physiotherapists, etc) as part of the patients’ care teams.

16. Overall comment on the discussion is to be careful not to repeat all the results – focus on few significant results and put them in perspective.

Conclusion

17. Lines 325-328: this sentence is too long and too vague. Focus on the key message of this scoping review. What do you want people to know? What is the main thing we should focus on/study after reading your scoping review?

**********

what does this mean?). If published, this will include your full peer review and any attached files.). If published, this will include your full peer review and any attached files.). If published, this will include your full peer review and any attached files.). If published, this will include your full peer review and any attached files.

If you choose “no”, your identity will remain anonymous but your review may still be made public.

**Do you want your identity to be public for this peer review?** For information about this choice, including consent withdrawal, please see our For information about this choice, including consent withdrawal, please see our For information about this choice, including consent withdrawal, please see our For information about this choice, including consent withdrawal, please see our Privacy Policy..

Reviewer #1: No

Reviewer #2: No

**********

[NOTE: If reviewer comments were submitted as an attachment file, they will be attached to this email and accessible via the submission site. Please log into your account, locate the manuscript record, and check for the action link "View Attachments". If this link does not appear, there are no attachment files.]

To ensure your figures meet our technical requirements, please review our figure guidelines: https://journals.plos.org/plosone/s/figures

You may also use PLOS’s free figure tool, NAAS, to help you prepare publication quality figures: https://journals.plos.org/plosone/s/figures#loc-tools-for-figure-preparation.

NAAS will assess whether your figures meet our technical requirements by comparing each figure against our figure specifications.

# Author comment: Author response to Decision Letter 1

**DOI:** 10.1371/journal.pone.0347295.r002

11 Nov 2025

Dear Editor,

Thank you very much for your letter and editor's positive feedback on our manuscript entitled “Unmet supportive care needs of head and neck cancer survivors: A scoping review” (Manuscript ID: PONE-D-25-46612). We appreciate the opportunity to revise the manuscript and address editor's comments. According to editor's request, we have carefully revised the manuscript and point-by-point addressed the academic editor and reviewers’ concerns. All revisions made in ‘Revised Manuscript with Track Changes’ have been highlighted in yellow and annotated in the revised manuscript. We hope that our manuscript could be considered for possible acceptance for publication in PLOS ONE. The main corrections in the manuscript and the responds to the editor's comments are as follows:

Reviewer #1:

1. - A major part of the study includes evaluation of existing tools to screen unmet needs. This could be clarified in the abstract.

Response: We sincerely thank the reviewer#1 for this critical insight. The aim in the revised abstract has included the evaluation of existing tools to screen unmet needs. This change is reflected in the revised manuscript on line 2, page 2.

2. - Given the variety of studies (quantitative, qualitative, and mixed methodologies), could the authors comment and include whether any of the qualitative or mixed studies found any domains of supportive needs that were not identified in the evaluation tools?

Response: We thank the reviewer#1 for this valuable suggestion. The Results and discussion sections now include findings on the domains of supportive needs found through qualitative and mixed-methods research, which were not identified by the evaluation tools. The change is reflected in the revised manuscript on line116-119/127-128/137, page 10 and line 240-244, page 15.

3. - I found Table 3 is thorough though a bit hard to digest as a reader. Would consider consolidating the prose or reformatting to consolidate the table.

Response: We sincerely thank the reviewer#1 for this valuable feedback. Table 3 has been consolidated and reformatted to improve readability and is now presented as Table 2. Please see the changes in the revised manuscript on line 105-107, page 8-9.

4. - In the limitations, would also consider including that cultural aspects are not included in these studies extracted. All studies are from high-income North American and European countries. Cultural elements from other high-income countries include from other LMICs are not included and may influence unmet social needs.

Response: We thank the reviewer#1 for raising this critical point. We have added a point about cultural aspects to the limitations section. Please see the changes in the revised manuscript on line 276-278, page 17.

Reviewer #2:

Introduction

1. In the first sentence of the article, the authors mention that head and neck cancers are the 6th most common type of malignancy. However, this epidemiologic definition usually only includes cancers of the mucosal lining including sinus, nasopharynx, oral and hypopharynx, larynx, oral cavity and sometimes salivary glands. Cutaneous malignancies and thyroid cancers are excluded from this definition.

Response: We thank the reviewer#2 for this critical clarification. The first sentence of the introduction has been revised to align with the standard epidemiological definition. The text now clearly states that our review focuses on cancers of the sinus, nasopharynx, oro and hypopharynx, larynx, oral cavity and salivary glands. Please see the changes in the revised manuscript on line 18-19, page 3.

2. From line 27-37, the authors describe what type of complications patients may experience and I have several issues with how this is phrased:

- The sentence “While life-saving, treatments frequently incur substantial adverse effects that generate persistent multidimensional burdens for HNC survivors throughout post-treatment survivorship” is extremely vague and lacks direction. I would suggest a broad description of how patients are affected “head and neck cancer treatment will have a significant impact on patients’ appearances as well as breathing & eating function […].

- Some specific side effects of treatment are mentioned with the exclusion of surgical treatment. I would advise that the authors mention all modalities of treatment with their main side effects without being too granular. The focus should be on radiation therapy and surgery as these are the modalities with the most impact.

Response: -We thank the reviewer#2 for this suggestion. The vague sentence has been replaced with a more direct description of the impact on appearance, breathing, and eating, as recommended. Please see the changes in the revised manuscript on line 27-30, page 3.

-We thank the reviewer#2 for this valuable suggestion. The relevant paragraph has been revised to mention all treatment modalities and their main side effects (without being too granular), with primary emphasis on radiation therapy and surgery. Please see the changes in the revised manuscript on line 30-34, page 3.

Review questions

3. I would like the first question to be “what are the unmet needs” and the second question to be “what tools are used.” This order makes it more like “what do we know so far” and “how is it usually measured/are we missing anything”

Response: We thank the reviewer#2 for this suggestion. The review questions have been reordered accordingly, addressing "what are the unmet needs" first, followed by "what tools are used.". Please see the changes in the revised manuscript on line 54-55, page 4.

Inclusion and exclusion criteria

4. I believe inclusion and exclusion criteria should be written as sentences and not point form “we included…” “we excluded …”

Response: We thank the reviewer#2 for this suggestion. The inclusion and exclusion criteria have been reformatted from bullet points into a narrative paragraph. Please see the updated text on line 63-70, page 5.

5. For the scoping review to be coherent in terms of patient population, I would include only aerodigestive tract cancers (as mentioned in my first comment). Cutaneous and thyroid malignancies should be excluded from the review as they have completely different prognoses and treatment profiles.

Response: We thank the reviewer#2 for reinforcing this critical point. The inclusion criteria have been revised to specify that only patients with aerodigestive tract cancers (originating from the sinus, nasopharynx, oro and hypopharynx, larynx, oral cavity and salivary glands) were included, while those with cutaneous or thyroid malignancies were excluded. This change has been made in the methods section on line 64-65, page 5.

Data synthesis

6. This paragraph was a bit unclear to me. Studies presented their own tool, but your reclassified according to the SCNS-SF34? This section needs to be clarified.

Response: We sincerely thank the reviewer#2 for raising this point. The paragraph has been revised to clarify that we applied the pre-existing frameworks of the SCNS-SF34 and SCNS-HNC to synthesize and categorize the unmet needs reported across studies. This change has been made in the methods section on line 80-87, page 6.

Results

7. The types of cancers in each study should be mentioned in table 1.

Response: We thank the reviewer#2 for this suggestion. The types of HNC for each included study have now been added to Table 1. This change has been made on line 96, page 7.

8. Again, as for the review questions, I would order the actual unmet needs results before what tools were used.

Response: We thank the reviewer#2 for this suggestion. The results have been reordered to present the unmet needs before the description of the assessment tools. The change has been made in the methods section on line 98-170, page 8-13.

9. Line 123-124: Health system/information, psychological, patient care/support needs were the top 3 unmet needs. **These were the results you find in your review, it does not mean it is the truth.

Response: We thank the reviewer#2 for this comment. The sentence has been revised to clarify that the identified top 3 unmet needs are based on the synthesis of the included studies. Please see the updated sentence on line 101-103, page 8.

10. I think table 4 should be removed and kept as the paragraph describing what are the main factors that were found to influence unmet supportive care needs. The table is a bit confusing as is.

Response: We thank the reviewer#2 for this suggestion. As recommended, we have removed Table 4 and integrated some paragraphs describing the main factors that were found to influence unmet supportive care needs. Please see the updated sentence on line 171-208, page 13-14.

11. At line 220, there is a mention of thyroid cancer patients. I strongly believe this cancer population should not be included in the study.

Response: We thank the reviewer#2 for this insightful comment. We have now systematically removed all mentions of thyroid cancer patients throughout the manuscript to ensure consistency with our focused scope on HNC. Please see the updated sentence on line 188-189, page 13.

Discussion

12. Lines 242-246: these lines should be removed, no need to justify the study at the start of the discussion. A discussion should start with what the study was and what the main finding was. “In our scoping review, we found that the 3 most frequently unmet supportive care needs were […]”

Response: We thank the reviewer#2 for this guidance. The opening paragraph of the discussion now directly presents the primary results, beginning with: "In our scoping review, we found that the 3 most frequently unmet supportive care needs were...". Please see the updated sentence on line 210-212, page 14.

13. Again, start by describing unmet needs and then go on with what tools were used.

Response: We thank the reviewer#2 for this suggestion. The sentences in the discussion have been reordered to first describe the unmet needs and then address the tools used. Please see the updated sentence on line 210-249, page 14-16.

14. Line 262: authors report a high willingness to pay and refer to a qualitative study that inherently has very few patients/patients willing to participate in research studies. This is likely a biased sample and I would refrain from mentioning this in the discussion. A significant portion of HNC patients come from a lower sociodemographics background and it would be hard for any head & neck oncologist to believe their patients have the funds/willingness to pay out of pocket for psychology services.

Response: We thank the reviewer#2 for this suggestion. The mention of high willingness to pay has been removed from the discussion. Please see the updated sentence on line 210-233, page 14-15.

15. Lines 274-279: I agree with the authors that multidisciplinarity helps with the care of patients and survivors. I would go further and mention the impact of nurse navigators and other allied health professionals (SLP, dietician, physiotherapists, etc) as part of the patients’ care teams.

Response: We sincerely thank the reviewer#2 for this suggestion. We have expanded the discussion to explicitly include the impact of nurse navigators and other allied health professionals such as speech-language pathologists, dietitians, and physiotherapists. Please see the updated sentence on line 224-229, page 15.

16. Overall comment on the discussion is to be careful not to repeat all the results – focus on few significant results and put them in perspective.

Response: We thank the reviewer#2 for this critical guidance. The discussion has been revised to focus on interpreting the key findings and their implications, rather than repeating the results. (line 212-223/250-264, page 14-16)

Conclusion

17. Lines 325-328: this sentence is too long and too vague. Focus on the key message of this scoping review. What do you want people to know? What is the main thing we should focus on/study after reading your scoping review?

Response: We thank the reviewer#2 for this constructive feedback. The conclusion has been revised to present a clearer and more focused key message, emphasizing the need for multidisciplinary care teams and comprehensive assessment tools. (line 286-289, page 17)

We tried our best to improve the manuscript and made some changes in the manuscript. These changes will not influence the primary content and framework of the manuscript. Furthermore, we confirm that the manuscript has been revised to meet PLOS ONE's style requirements using the provided templates. The Data Availability Statement has been updated to include the repository name. Captions for Supporting Information files have been added at the end of the manuscript, with all in-text citations updated accordingly.

We appreciate for editor's warm work earnestly, and hope that the correction will meet with approval. Once again, thank you very much for your comments and suggestions.

Sincerely yours,

Gu-Qing Zeng, Ph.D. Professor and Director

School of Nursing, Hengyang Medical School, University of South China, Hengyang 421001, P.R. China

Tel: 86-734-8281821

Fax: 86-734-8281344

E-mail: zengguqing0123@163.com

Attachment Submitted filename: Response to Reviewers.docx

# Peer review: Decision Letter 1

**DOI:** 10.1371/journal.pone.0347295.r003

**Authors:** Jen Edwards

1 Dec 2025

Dear Dr. Zeng,

Thank you for submitting your manuscript to PLOS ONE. After careful consideration, we feel that it has merit but does not fully meet PLOS ONE’s publication criteria as it currently stands. Therefore, we invite you to submit a revised version of the manuscript that addresses the points raised during the review process.

Please submit your revised manuscript by Jan 15 2026 11:59PM. If you will need more time than this to complete your revisions, please reply to this message or contact the journal office at plosone@plos.org. When you're ready to submit your revision, log on to https://www.editorialmanager.com/pone/ and select the 'Submissions Needing Revision' folder to locate your manuscript file.. When you're ready to submit your revision, log on to https://www.editorialmanager.com/pone/ and select the 'Submissions Needing Revision' folder to locate your manuscript file.. When you're ready to submit your revision, log on to https://www.editorialmanager.com/pone/ and select the 'Submissions Needing Revision' folder to locate your manuscript file.. When you're ready to submit your revision, log on to https://www.editorialmanager.com/pone/ and select the 'Submissions Needing Revision' folder to locate your manuscript file.

- A rebuttal letter that responds to each point raised by the academic editor and reviewer(s). You should upload this letter as a separate file labeled 'Response to Reviewers'.
- A marked-up copy of your manuscript that highlights changes made to the original version. You should upload this as a separate file labeled 'Revised Manuscript with Track Changes'.
- An unmarked version of your revised paper without tracked changes. You should upload this as a separate file labeled 'Manuscript'.

If applicable, we recommend that you deposit your laboratory protocols in protocols.io to enhance the reproducibility of your results. Protocols.io assigns your protocol its own identifier (DOI) so that it can be cited independently in the future. For instructions see: https://journals.plos.org/plosone/s/submission-guidelines#loc-laboratory-protocols. Additionally, PLOS ONE offers an option for publishing peer-reviewed Lab Protocol articles, which describe protocols hosted on protocols.io. Read more information on sharing protocols at . Additionally, PLOS ONE offers an option for publishing peer-reviewed Lab Protocol articles, which describe protocols hosted on protocols.io. Read more information on sharing protocols at . Additionally, PLOS ONE offers an option for publishing peer-reviewed Lab Protocol articles, which describe protocols hosted on protocols.io. Read more information on sharing protocols at . Additionally, PLOS ONE offers an option for publishing peer-reviewed Lab Protocol articles, which describe protocols hosted on protocols.io. Read more information on sharing protocols at https://plos.org/protocols?utm_medium=editorial-email&utm_source=authorletters&utm_campaign=protocols....

We look forward to receiving your revised manuscript.

Kind regards,

Jen Edwards

Staff Editor

PLOS ONE

Journal Requirements:

If the reviewer comments include a recommendation to cite specific previously published works, please review and evaluate these publications to determine whether they are relevant and should be cited. There is no requirement to cite these works unless the editor has indicated otherwise.

Please review your reference list to ensure that it is complete and correct. If you have cited papers that have been retracted, please include the rationale for doing so in the manuscript text, or remove these references and replace them with relevant current references. Any changes to the reference list should be mentioned in the rebuttal letter that accompanies your revised manuscript. If you need to cite a retracted article, indicate the article’s retracted status in the References list and also include a citation and full reference for the retraction notice.

Additional Editor Comments:

There are a number of remaining concerns which must be addressed before your manuscript can be accepted for publication. Firstly, it is unclear why only 434 records were screened out of 2230 retrieved. Please update your PRISMA diagram to identify the 1796 papers that were removed before screening and indicate why these were removed.

Secondly, please note that PLOS ONE does not copyedit accepted manuscripts and that one of our criteria for publication is that articles must be presented in an intelligible fashion and written in clear, correct, and unambiguous English (https://journals.plos.org/plosone/s/criteria-for-publication#loc-5). We suggest you have a fluent English-language speaker thoroughly copyedit your manuscript for language usage, spelling, and grammar. For example, there are a number of inappropriate uses of 'etc.' in various places (such as lines 4, 66, 134, 150, 242). A second example is that 'two reviewers' should be used in preference to '2 reviewers'. Please note that these examples are not exhaustive.

If you do not know anyone who can do this, you may wish to consider employing a professional scientific editing service. While you may approach any qualified individual or any professional scientific editing service of your choice, PLOS has partnered with American Journal Experts (AJE) to provide discounted services to PLOS authors. AJE has extensive experience helping authors meet PLOS guidelines and can provide language editing, translation, manuscript formatting, and figure formatting to ensure your manuscript meets our submission guidelines. If the PLOS editorial team finds any language issues in text that AJE has edited, AJE will re-edit the text for free. To take advantage of this special partnership, use the following link: https://www.aje.com/go/plos/. Please note that PLOS does not financially benefit from this partnership; moreover, having the manuscript copyedited by AJE or any other editing services does not guarantee selection for peer review.

[Note: HTML markup is below. Please do not edit.]

Reviewers' comments:

Reviewer's Responses to Questions

**Comments to the Author**

Reviewer #1: All comments have been addressed

Reviewer #2: All comments have been addressed

**********

2. Is the manuscript technically sound, and do the data support the conclusions?

Reviewer #1: Yes

Reviewer #2: Yes

**********

3. Has the statistical analysis been performed appropriately and rigorously?

Reviewer #1: N/A

Reviewer #2: Yes

**********

4. Have the authors made all data underlying the findings in their manuscript fully available?

The PLOS Data policy requires authors to make all data underlying the findings described in their manuscript fully available without restriction, with rare exception (please refer to the Data Availability Statement in the manuscript PDF file). The data should be provided as part of the manuscript or its supporting information, or deposited to a public repository. For example, in addition to summary statistics, the data points behind means, medians and variance measures should be available. If there are restrictions on publicly sharing data—e.g. participant privacy or use of data from a third party—those must be specified.requires authors to make all data underlying the findings described in their manuscript fully available without restriction, with rare exception (please refer to the Data Availability Statement in the manuscript PDF file). The data should be provided as part of the manuscript or its supporting information, or deposited to a public repository. For example, in addition to summary statistics, the data points behind means, medians and variance measures should be available. If there are restrictions on publicly sharing data—e.g. participant privacy or use of data from a third party—those must be specified.

Reviewer #1: Yes

Reviewer #2: Yes

**********

5. Is the manuscript presented in an intelligible fashion and written in standard English?

Reviewer #1: Yes

Reviewer #2: Yes

**********

Reviewer #1: Thank you to the authors for their responses in addressing the recommendations. I feel the authors have addressed the edits well and thoroughly.

Reviewer #2: I want to congratulate the authors for undertaking this extensive work. I was satisfied with all the reviews made in the article.

**********

what does this mean?). If published, this will include your full peer review and any attached files.). If published, this will include your full peer review and any attached files.). If published, this will include your full peer review and any attached files.). If published, this will include your full peer review and any attached files.

If you choose “no”, your identity will remain anonymous but your review may still be made public.

**Do you want your identity to be public for this peer review?** For information about this choice, including consent withdrawal, please see our For information about this choice, including consent withdrawal, please see our For information about this choice, including consent withdrawal, please see our For information about this choice, including consent withdrawal, please see our Privacy Policy..

Reviewer #1: No

Reviewer #2: No

**********

[NOTE: If reviewer comments were submitted as an attachment file, they will be attached to this email and accessible via the submission site. Please log into your account, locate the manuscript record, and check for the action link "View Attachments". If this link does not appear, there are no attachment files.]

To ensure your figures meet our technical requirements, please review our figure guidelines: https://journals.plos.org/plosone/s/figures

You may also use PLOS’s free figure tool, NAAS, to help you prepare publication quality figures: https://journals.plos.org/plosone/s/figures#loc-tools-for-figure-preparation.

NAAS will assess whether your figures meet our technical requirements by comparing each figure against our figure specifications.

# Author comment: Author response to Decision Letter 2

**DOI:** 10.1371/journal.pone.0347295.r004

5 Dec 2025

Dear Editor,

Thank you very much for your letter and editor's positive feedback on our manuscript entitled “Unmet supportive care needs of head and neck cancer survivors: A scoping review” (Manuscript ID: PONE-D-25-46612). We appreciate the opportunity to revise the manuscript and address editor's comments. According to editor's request, we have carefully revised the manuscript and point-by-point addressed the academic editor’s concerns. All revisions made in ‘Revised Manuscript with Track Changes’ have been highlighted in yellow in the revised manuscript. We hope that our manuscript could be considered for possible acceptance for publication in PLOS ONE. The main corrections in the manuscript and the responds to the editor's comments are as follows:

- Firstly, it is unclear why only 434 records were screened out of 2230 retrieved. Please update your PRISMA diagram to identify the 1796 papers that were removed before screening and indicate why these were removed.

Response: Thank you for your valuable feedback. We have carefully updated our PRISMA flow diagram in accordance with your comment. The updated figure file has been uploaded to the submission system.

- Secondly, please note that PLOS ONE does not copyedit accepted manuscripts and that one of our criteria for publication is that articles must be presented in an intelligible fashion and written in clear, correct, and unambiguous English (https://journals.plos.org/plosone/s/criteria-for-publication#loc-5). We suggest you have a fluent English-language speaker thoroughly copyedit your manuscript for language usage, spelling, and grammar. For example, there are a number of inappropriate uses of 'etc.' in various places (such as lines 4, 66, 134, 150, 242). A second example is that 'two reviewers' should be used in preference to '2 reviewers'. Please note that these examples are not exhaustive.

Response: Thank you for your valuable feedback on language quality. We have thoroughly revised the manuscript in response to your suggestions and have had the entire text copyedited by a fluent English-language speaker to ensure clarity and correctness. In addition, all specific content mentioned in your comments are available in Line 3-4, 64, 79, 134, 147-149 and 240-241.

Following the editorial guidance, we have carefully reviewed all comments. We confirm that no reviewer suggestions required citing additional works, and we have verified the completeness and accuracy of the reference list, including confirming that no retracted articles are cited.

We appreciate for editor's warm work earnestly, and hope that the correction will meet with approval. Once again, thank you very much for your comments and suggestions.

Sincerely yours,

Gu-Qing Zeng, Ph.D. Professor and Director

School of Nursing, Hengyang Medical School, University of South China, Hengyang 421001, P.R. China

Tel: 86-734-8281821

Fax: 86-734-8281344

E-mail: zengguqing0123@163.com

Attachment Submitted filename: Response_to_Reviewers_auresp_2.docx

# Peer review: Decision Letter 2

**DOI:** 10.1371/journal.pone.0347295.r005

**Authors:** Enes Erul

22 Feb 2026

Dear Dr. Zeng,

Thank you for your submission to PLOS One. The editors have completed their review of your manuscript. I am pleased to inform you that they found your manuscript potentially acceptable for publication in PLOS One provided you address the reviewers' and editors' comments.

In order to expedite final decision-making, we ask that you submit your revised manuscript by Apr 08 2026 11:59PM.  See below for information about submitting your manuscript.

plosone@plos.org. When you're ready to submit your revision, log on to https://www.editorialmanager.com/pone/ and select the 'Submissions Needing Revision' folder to locate your manuscript file.. When you're ready to submit your revision, log on to https://www.editorialmanager.com/pone/ and select the 'Submissions Needing Revision' folder to locate your manuscript file.. When you're ready to submit your revision, log on to https://www.editorialmanager.com/pone/ and select the 'Submissions Needing Revision' folder to locate your manuscript file.. When you're ready to submit your revision, log on to https://www.editorialmanager.com/pone/ and select the 'Submissions Needing Revision' folder to locate your manuscript file.

- A letter that responds to each point raised by the academic editor and reviewer(s). You should upload this letter as a separate file labeled 'Response to Reviewers'.
- A marked-up copy of your manuscript that highlights changes made to the original version. You should upload this as a separate file labeled 'Revised Manuscript with Track Changes'.
- An unmarked version of your revised paper without tracked changes. You should upload this as a separate file labeled 'Manuscript'.

If applicable, we recommend that you deposit your laboratory protocols in protocols.io to enhance the reproducibility of your results. Protocols.io assigns your protocol its own identifier (DOI) so that it can be cited independently in the future. For instructions see: https://journals.plos.org/plosone/s/submission-guidelines#loc-laboratory-protocols. Additionally, PLOS ONE offers an option for publishing peer-reviewed Lab Protocol articles, which describe protocols hosted on protocols.io. Read more information on sharing protocols at . Additionally, PLOS ONE offers an option for publishing peer-reviewed Lab Protocol articles, which describe protocols hosted on protocols.io. Read more information on sharing protocols at . Additionally, PLOS ONE offers an option for publishing peer-reviewed Lab Protocol articles, which describe protocols hosted on protocols.io. Read more information on sharing protocols at . Additionally, PLOS ONE offers an option for publishing peer-reviewed Lab Protocol articles, which describe protocols hosted on protocols.io. Read more information on sharing protocols at https://plos.org/protocols?utm_medium=editorial-email&utm_source=authorletters&utm_campaign=protocols....

We look forward to receiving your revised manuscript.

Kind regards,

Enes Erul, MD

Academic Editor

PLOS One

Journal Requirements:

If the reviewer comments include a recommendation to cite specific previously published works, please review and evaluate these publications to determine whether they are relevant and should be cited. There is no requirement to cite these works unless the editor has indicated otherwise.

Please review your reference list to ensure that it is complete and correct. If you have cited papers that have been retracted, please include the rationale for doing so in the manuscript text, or remove these references and replace them with relevant current references. Any changes to the reference list should be mentioned in the rebuttal letter that accompanies your revised manuscript. If you need to cite a retracted article, indicate the article’s retracted status in the References list and also include a citation and full reference for the retraction notice.

[Note: HTML markup is below. Please do not edit.]

Reviewers' comments:

Reviewer's Responses to Questions

**Comments to the Author**

Reviewer #1: All comments have been addressed

Reviewer #2: All comments have been addressed

Reviewer #3: (No Response)

**********

2. Is the manuscript technically sound, and do the data support the conclusions?

Reviewer #1: Yes

Reviewer #2: Yes

Reviewer #3: Yes

**********

3. Has the statistical analysis been performed appropriately and rigorously?

Reviewer #1: N/A

Reviewer #2: Yes

Reviewer #3: N/A

**********

4. Have the authors made all data underlying the findings in their manuscript fully available?

The PLOS Data policy requires authors to make all data underlying the findings described in their manuscript fully available without restriction, with rare exception (please refer to the Data Availability Statement in the manuscript PDF file). The data should be provided as part of the manuscript or its supporting information, or deposited to a public repository. For example, in addition to summary statistics, the data points behind means, medians and variance measures should be available. If there are restrictions on publicly sharing data—e.g. participant privacy or use of data from a third party—those must be specified.requires authors to make all data underlying the findings described in their manuscript fully available without restriction, with rare exception (please refer to the Data Availability Statement in the manuscript PDF file). The data should be provided as part of the manuscript or its supporting information, or deposited to a public repository. For example, in addition to summary statistics, the data points behind means, medians and variance measures should be available. If there are restrictions on publicly sharing data—e.g. participant privacy or use of data from a third party—those must be specified.

Reviewer #1: Yes

Reviewer #2: Yes

Reviewer #3: Yes

**********

5. Is the manuscript presented in an intelligible fashion and written in standard English?

Reviewer #1: Yes

Reviewer #2: Yes

Reviewer #3: Yes

**********

Reviewer #1: Thank you to the authors for their work. I feel as though this manuscript is suitable for publication and adds value to the existing literature.

Reviewer #2: The authors made commendable efforts to improve the manuscript after our reviews. I have no further comments on this final version of the paper.

Reviewer #3: This manuscript presents a scoping review examining unmet supportive care needs among head and neck cancer (HNC) survivors. The topic is clinically important and highly relevant given the complex functional, psychological, and informational challenges faced by this population. The manuscript is generally well organized, clearly structured, and aligned with PRISMA-ScR guidance. The categorization of unmet needs into seven domains provides a useful framework, and the summary of assessment tools contributes practical value to the field.

Overall, the study meets PLOS ONE’s criteria for technical soundness. However, several revisions would strengthen methodological transparency and improve conceptual precision.

1. Study Selection Transparency

While a PRISMA flow diagram is referenced, the manuscript would benefit from clearer reporting of the study selection process. Specifically:

The number of duplicates removed should be explicitly stated.

Reasons for full-text exclusions should be categorized and quantified.

A brief textual summary of the most common exclusion reasons in the Results section would enhance clarity and reproducibility.

2. Clarification of “Top Three” Unmet Needs

The manuscript identifies health system/information, patient care/support, and psychological needs as the “top three” unmet needs. However, it is unclear how this ranking was determined. Please clarify whether this classification is based on:

Frequency of reporting across included studies,

Number of unmet items,

Proportion of participants reporting these needs,

Or another defined metric.

Without an explicit operational definition, the phrasing may overstate the strength of the evidence.

3. Framing of Conclusions

As this is a scoping review, the primary aim is evidence mapping rather than causal inference or prescriptive recommendation. Some parts of the Discussion (e.g., recommendations regarding multidisciplinary team expansion and telehealth integration) should be more clearly framed as implications or future directions rather than conclusions directly derived from the data.

Additionally, the statement that this study provides the “first categorization and integration” of unmet needs among HNC survivors should be qualified unless clearly supported by the literature.

4. Assessment Tool Discussion

The comparison of supportive care needs assessment instruments is a valuable contribution. However, critiques regarding tool “incompleteness” are presented without formal psychometric evaluation. If assessment of validity, reliability, or measurement quality was beyond the scope of this review, this should be clearly acknowledged in the Methods and/or Limitations section.

5. Influencing Factors

The section discussing demographic, clinical, and psychological correlates is informative but could be better organized. Consider grouping findings by unmet need domain and clarifying whether reported associations were adjusted or unadjusted in the original studies. Causal language should be avoided unless supported by longitudinal evidence.

One statement regarding education level and psychological needs appears inconsistent and should be clarified.

6. Language and Presentation

The manuscript is generally written in clear academic English. Minor revisions are needed to:

Replace informal phrasing (e.g., “lots of unmet needs”).

Standardize terminology across need domains.

Correct spacing issues (e.g., “HNC-specific” formatting).

Improve conciseness in parts of the Introduction and Discussion.

No concerns regarding dual publication or research ethics are apparent.

In summary, this is a well-structured and clinically meaningful scoping review that would be suitable for publication after minor to moderate revision addressing the points above.

**********

what does this mean?). If published, this will include your full peer review and any attached files.). If published, this will include your full peer review and any attached files.). If published, this will include your full peer review and any attached files.). If published, this will include your full peer review and any attached files.

If you choose “no”, your identity will remain anonymous but your review may still be made public.

**Do you want your identity to be public for this peer review?** For information about this choice, including consent withdrawal, please see our For information about this choice, including consent withdrawal, please see our For information about this choice, including consent withdrawal, please see our For information about this choice, including consent withdrawal, please see our Privacy Policy..

Reviewer #1: No

Reviewer #2: **Yes:** Noémie Villemure-PoliquinNoémie Villemure-PoliquinNoémie Villemure-PoliquinNoémie Villemure-Poliquin

Reviewer #3: **Yes:** Imene HadjiImene HadjiImene HadjiImene Hadji

**********

[NOTE: If reviewer comments were submitted as an attachment file, they will be attached to this email and accessible via the submission site. Please log into your account, locate the manuscript record, and check for the action link "View Attachments". If this link does not appear, there are no attachment files.]

To ensure your figures meet our technical requirements, please review our figure guidelines: https://journals.plos.org/plosone/s/figures

You may also use PLOS’s free figure tool, NAAS, to help you prepare publication quality figures: https://journals.plos.org/plosone/s/figures#loc-tools-for-figure-preparation.

NAAS will assess whether your figures meet our technical requirements by comparing each figure against our figure specifications.

# Author comment: Author response to Decision Letter 3

**DOI:** 10.1371/journal.pone.0347295.r006

21 Mar 2026

Dear Editor,

Thank you very much for your letter and editor's positive feedback on our manuscript entitled “Unmet supportive care needs of head and neck cancer survivors: A scoping review” (Manuscript ID: PONE-D-25-46612). We appreciate the opportunity to revise the manuscript and address reviewers' comments. According to editor's request, we have carefully revised the manuscript and point-by-point addressed the reviewers’ concerns. All revisions made in ‘Revised Manuscript with Track Changes’ have been highlighted in yellow and annotated in the revised manuscript. We hope that our manuscript could be considered for possible acceptance for publication in PLOS ONE. The main corrections in the manuscript are as follows:

Reviewer #3:

1. Study Selection Transparency

While a PRISMA flow diagram is referenced, the manuscript would benefit from clearer reporting of the study selection process. Specifically:

The number of duplicates removed should be explicitly stated.

Reasons for full-text exclusions should be categorized and quantified.

A brief textual summary of the most common exclusion reasons in the Results section would enhance clarity and reproducibility.

Response: We sincerely thank the reviewer#3 for this critical insight. We have revised the manuscript accordingly. These changes are reflected in the revised manuscript on line 87-91, page 6.

2. Clarification of “Top Three” Unmet Needs

The manuscript identifies health system/information, patient care/support, and psychological needs as the “top three” unmet needs. However, it is unclear how this ranking was determined. Please clarify whether this classification is based on:

Frequency of reporting across included studies,

Number of unmet items,

Proportion of participants reporting these needs,

Or another defined metric.

Without an explicit operational definition, the phrasing may overstate the strength of the evidence.

Response: We sincerely thank the reviewer#3 for this critical insight. We have revised the manuscript accordingly. This change is reflected in the revised manuscript on line 102-104, page 8.

3. Framing of Conclusions

As this is a scoping review, the primary aim is evidence mapping rather than causal inference or prescriptive recommendation. Some parts of the Discussion (e.g., recommendations regarding multidisciplinary team expansion and telehealth integration) should be more clearly framed as implications or future directions rather than conclusions directly derived from the data.

Additionally, the statement that this study provides the “first categorization and integration” of unmet needs among HNC survivors should be qualified unless clearly supported by the literature.

Response: We sincerely thank the reviewer#3 for this critical insight. We have revised the Discussion section accordingly. This change is reflected in the revised manuscript on line 224-225/230-231/235-237/267-268, page 15.

4. Assessment Tool Discussion

The comparison of supportive care needs assessment instruments is a valuable contribution. However, critiques regarding tool “incompleteness” are presented without formal psychometric evaluation. If assessment of validity, reliability, or measurement quality was beyond the scope of this review, this should be clearly acknowledged in the Methods and/or Limitations section.

Response: We sincerely thank the reviewer#3 for this critical insight. To address this concern, we have revised the Limitations section. This change is reflected in the revised manuscript on line 282-283, page 17.

5. Influencing Factors

The section discussing demographic, clinical, and psychological correlates is informative but could be better organized. Consider grouping findings by unmet need domain and clarifying whether reported associations were adjusted or unadjusted in the original studies. Causal language should be avoided unless supported by longitudinal evidence.

One statement regarding education level and psychological needs appears inconsistent and should be clarified.

Response: We sincerely thank the reviewer#3 for this critical insight. We have substantially revised this section to address your suggestions. Detailed changes can be found on line 171-210, page 13. Specifically, the revision about the statement regarding education level can be found on line 189-192, page 13-14.

6. Language and Presentation

The manuscript is generally written in clear academic English. Minor revisions are needed to:

Replace informal phrasing (e.g., “lots of unmet needs”)

Standardize terminology across need domains.

Correct spacing issues (e.g., “HNC-specific” formatting).

Improve conciseness in parts of the Introduction and Discussion.

No concerns regarding dual publication or research ethics are apparent.

Response: We sincerely thank the reviewer#3 for this critical insight. We have carefully revised the manuscript according to your suggestions. Specifically, informal phrasing (e.g., "lots of unmet needs") has been replaced with more formal academic expressions. These changes can be found on line 13, page 2.

We appreciate for editor's warm work earnestly, and hope that the correction will meet with approval. Once again, thank you very much for your comments and suggestions.

Sincerely yours,

Gu-Qing Zeng, Ph.D. Professor and Director

School of Nursing, Hengyang Medical School, University of South China, Hengyang 421001, P.R. China

Tel: 86-734-8281821

Fax: 86-734-8281344

E-mail: zengguqing0123@163.com

Attachment Submitted filename: Response_to_Reviewers_auresp_3.docx

# Editor report: Decision Letter 3

**DOI:** 10.1371/journal.pone.0347295.r007

**Authors:** Enes Erul

1 Apr 2026

Unmet supportive care needs of head and neck cancer survivors: A scoping review

PONE-D-25-46612R3

Dear Dr. Gu-qing Zeng,

We’re pleased to inform you that your manuscript has been judged scientifically suitable for publication and will be formally accepted for publication once it meets all outstanding technical requirements.

Within one week, you’ll receive an e-mail detailing the required amendments. When these have been addressed, you’ll receive a formal acceptance letter and your manuscript will be scheduled for publication.

An invoice will be generated when your article is formally accepted. Please note, if your institution has a publishing partnership with PLOS and your article meets the relevant criteria, all or part of your publication costs will be covered. Please make sure your user information is up-to-date by logging into Editorial Manager at Editorial Manager® and clicking the ‘Update My Information' link at the top of the page. For questions related to billing, please contact  and clicking the ‘Update My Information' link at the top of the page. For questions related to billing, please contact  and clicking the ‘Update My Information' link at the top of the page. For questions related to billing, please contact  and clicking the ‘Update My Information' link at the top of the page. For questions related to billing, please contact billing support....

If your institution or institutions have a press office, please notify them about your upcoming paper to help maximize its impact. If they’ll be preparing press materials, please inform our press team as soon as possible -- no later than 48 hours after receiving the formal acceptance. Your manuscript will remain under strict press embargo until 2 pm Eastern Time on the date of publication. For more information, please contact onepress@plos.org.

Kind regards,

Enes Erul, MD

Academic Editor

PLOS One

# Editor report: Acceptance letter

**DOI:** 10.1371/journal.pone.0347295.r008

**Authors:** Enes Erul

PONE-D-25-46612R3

PLOS One

Dear Dr. Zeng,

I'm pleased to inform you that your manuscript has been deemed suitable for publication in PLOS One. Congratulations! Your manuscript is now being handed over to our production team.

At this stage, our production department will prepare your paper for publication. This includes ensuring the following:

* All references, tables, and figures are properly cited

* All relevant supporting information is included in the manuscript submission,

* There are no issues that prevent the paper from being properly typeset

You will receive further instructions from the production team, including instructions on how to review your proof when it is ready. Please keep in mind that we are working through a large volume of accepted articles, so please give us a few days to review your paper and let you know the next and final steps.

Lastly, if your institution or institutions have a press office, please let them know about your upcoming paper now to help maximize its impact. If they'll be preparing press materials, please inform our press team within the next 48 hours. Your manuscript will remain under strict press embargo until 2 pm Eastern Time on the date of publication. For more information, please contact onepress@plos.org.

You will receive an invoice from PLOS for your publication fee after your manuscript has reached the completed accept phase. If you receive an email requesting payment before acceptance or for any other service, this may be a phishing scheme. Learn how to identify phishing emails and protect your accounts at https://explore.plos.org/phishing.

If we can help with anything else, please email us at customercare@plos.org.

Thank you for submitting your work to PLOS ONE and supporting open access.

Kind regards,

PLOS ONE Editorial Office Staff

on behalf of

Dr. Enes Erul

Academic Editor

PLOS One
